# Chimeric Ligands of Pili and Lectin A Inhibit Tolerance, Persistence, and Virulence Factors of *Pseudomonas aeruginosa* over a Wide Range of Phenotypes

**DOI:** 10.1021/acsinfecdis.2c00201

**Published:** 2022-06-06

**Authors:** Pankaj D. Patil, Hewen Zheng, Felicia N. Burns, Arizza C. S. Ibanez, Yuchen Jin, Yan-Yeung Luk

**Affiliations:** Department of Chemistry, Syracuse University, 1-014 Center of Science and Technology, Syracuse, New York 13244-4100, United States

**Keywords:** LecA, pili, pyocyanin, elastase production, P. aeruginosa

## Abstract

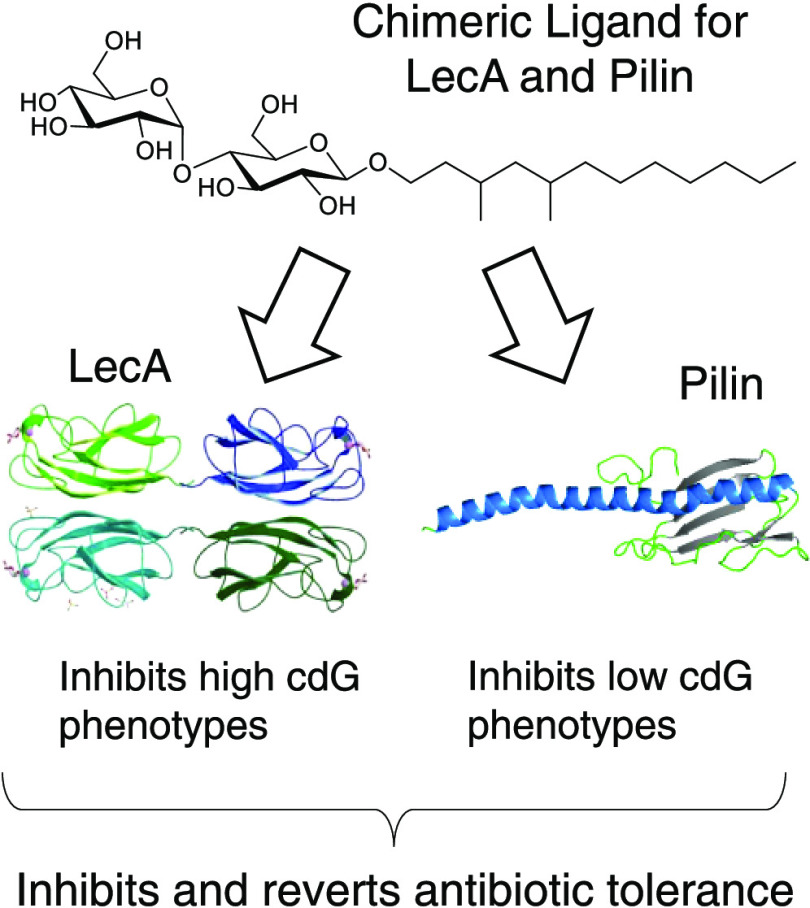

Bacteria readily form resilient phenotypes to counter environmental and antibiotic stresses. Here, we demonstrate a class of small molecules that inhibit a wide range of *Pseudomonas aeruginosa* phenotypes and enable antibiotics to kill previously tolerant bacteria, preventing the transition of tolerant bacteria into a persistent population. We identified two proteins, type IV pili and lectin LecA, as receptors for our molecules by methods including a new label-free assay based on bacterial motility sensing the chemicals in the environment, the chemical inhibition of bacteriophage adsorption on pili appendages of bacteria, and fluorescence polarization. Structure–activity relationship studies reveal a molecule that inhibits only pili appendage and a class of chimeric ligands that inhibit both LecA and pili. Important structural elements of the ligand are identified for each protein. This selective ligand binding identifies the phenotypes each protein receptor controls. Inhibiting LecA results in reducing biofilm formation, eliminating small colony variants, and is correlated with killing previously tolerant bacteria. Inhibiting pili appendages impedes swarming and twitching motilities and pyocyanin and elastase production. Because these phenotypes are controlled by a broad range of signaling pathways, this approach simultaneously controls the multiple signaling mechanisms preventing bacteria to elude antibiotic treatments.

Bacteria exhibit adaptive responses to a wide range of stresses from environmental cues, including antibiotic treatments. These responses result in the development of many resilient phenotypes,^1−19^ including augmented biofilm formation,^3−5^ enhanced motilities,^8,9^ surface attachment,^12−14^ chemical-induced virulence,^7,15,20^ and small colony variant (SCV) emergence.^16−18^ These diverse phenotypes are often controlled by or correlated with both high and low levels of the global small-molecule messenger, bis-(3′-5′)-cyclic dimeric guanosine monophosphate (cdG).^21−24^ For both in vitro and in vivo conditions, antibiotic treatments or host immune defense can trigger the increase in cdG levels in bacteria, leading to phenotypes that exhibit high drug tolerance and persistence, including biofilms,^3,19^ SCVs,^16−18^ and overexpression of extracellular polymeric substance (EPS).^25^

Recent studies revealed that low cdG phenotypes can also be highly virulent.^11,15,24^ Swarming bacteria, a low cdG phenotype, requires rhamnolipids^26−28^ and exhibit antibiotic resistance.^9,10^ Dispersing bacteria from biofilms by nitric oxide donor^15^ switches bacteria to low cdG phenotypes and causes increased production of virulence factors, including induction of type II and type III secretion systems, which has been shown to kill even macrophages.^15^ Surprisingly, under different conditions, the same antibiotic, tobramycin, can promote *Pseudomonas aeruginosa* to form either high or low cdG phenotypes to counter stresses. Sublethal doses of tobramycin promote the high cdG phenotypes, including increased biofilms and increased exopolymer production,^3^ and cause the formation of SCVs in vivo.^18^ In contrast, on a hydrated agar surface, tobramycin promotes the swarming motility of *P. aeruginosa*.^8^ These findings indicate that while the two types of protein domains, diguanylate cyclases and phosphodiesterases, that regulate the synthesis and hydrolysis of cdG are well established,^21,23^ their response to external stresses is still unpredictably complex. More importantly, these findings suggest that inhibiting high cdG phenotypes alone, such as biofilm formation, would not be sufficient to control drug tolerance and persistence as bacteria can readily transition between high and low cdG phenotypes.

In previous studies, we showed a class of disugar-derivatized hydrocarbons that inhibit both the biofilm formation and the swarming motility of *P. aeruginosa* bacteria.^29,30^ These dual inhibitors are intriguing as biofilm formation and swarming motility are controlled by opposite signaling pathways mediated by high and low cdG levels, respectively.^22^ Because bacteria can readily transition between both high and low cdG phenotypes, both of which can be virulent, this class of molecules provide opportunities to control all signaling that leads to drug tolerance, persistence, and resistance and also block bacterial signaling that escapes drug treatments by transitioning between phenotypes.

In this work, we demonstrate a specific class of molecules that inhibit a wide range of phenotypes that are associated with both high and low cdG levels. These phenotypes include the formation of biofilms, SCVs, pellicles, swarming and twitching motilities, and the production of virulence factors, including pyocyanin and elastase. Antibiotics such as tobramycin promote these phenotypes,^3,17,20^ which dwell in chronic and acute infections.^3^ We also demonstrate that this class of molecules, when combined with tobramycin, enable the killing of tolerant bacteria and prevent the formation of nascent persistent bacteria in biofilms.

To elucidate the mechanism behind this simultaneous control of a wide range of phenotypes associated with opposite signaling pathways (low and high cdG levels), we identify pili appendages and LecA as two protein receptors targeted by the same small molecule (Figure 1). We confirmed the binding of a small molecule to pili by multiple methods, including a novel bacterial motility-enabled binding assay, which is modified from the swarming experiment without any chemical labeling, and the small-molecule inhibition of pili-specific bacteriophage adsorption of *P. aeruginosa*. The LecA binding was characterized by the fluorescence polarization method and by the bacterial motility-enabled binding assay without chemical modification. Structure–activity studies identified the different structural elements of the small-molecule ligands that are important for each pilin and LecA protein, making this class of molecules a structural chimera. The selective ligand–receptor bindings of two proteins reveal the mapping between the proteins and the phenotypes they control.

**Figure 1:**
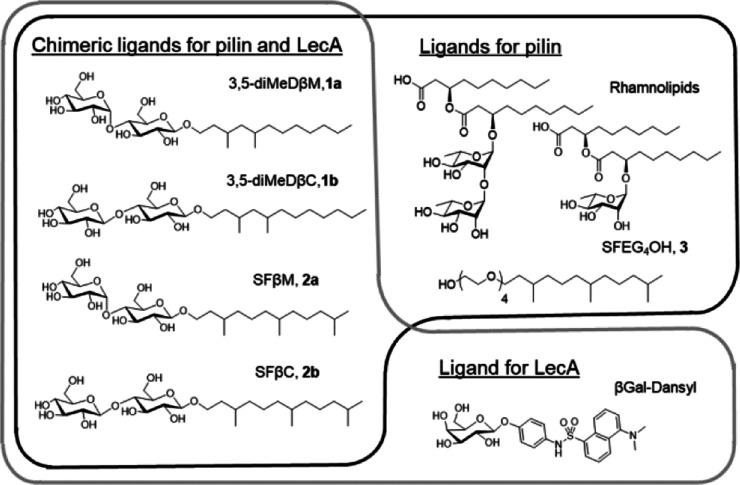
Venn diagram of chimeric ligand molecule structures for type IV pili and lectin LecA proteins and ligand molecules for only pilin and only LecA.

## Results and Discussion

Our previous studies reveal that the two most active molecules among these dual synthetic inhibitors are saturated farnesol-β-cellobioside and saturated farnesol-β-maltoside (SFβC and SFβM, Figure 1).^29^ Early structural studies indicate that methyl substitution in aliphatic chains is important; the linear C12-aliphatic chain does not exhibit strong bioactivity, whereas the 3,7,11-trimethyl-substituted aliphatic chain does.^29,30^ Searching for more potent molecules through the synthesis of structural variants, we explored new methyl positions on the C12-alphatic chain, resulting in new molecules: 3,5-diMeDβM, **1a**, and 3,5-diMeDβC, **1b** (Figures 2 and S16–S30).

**Figure 2:**
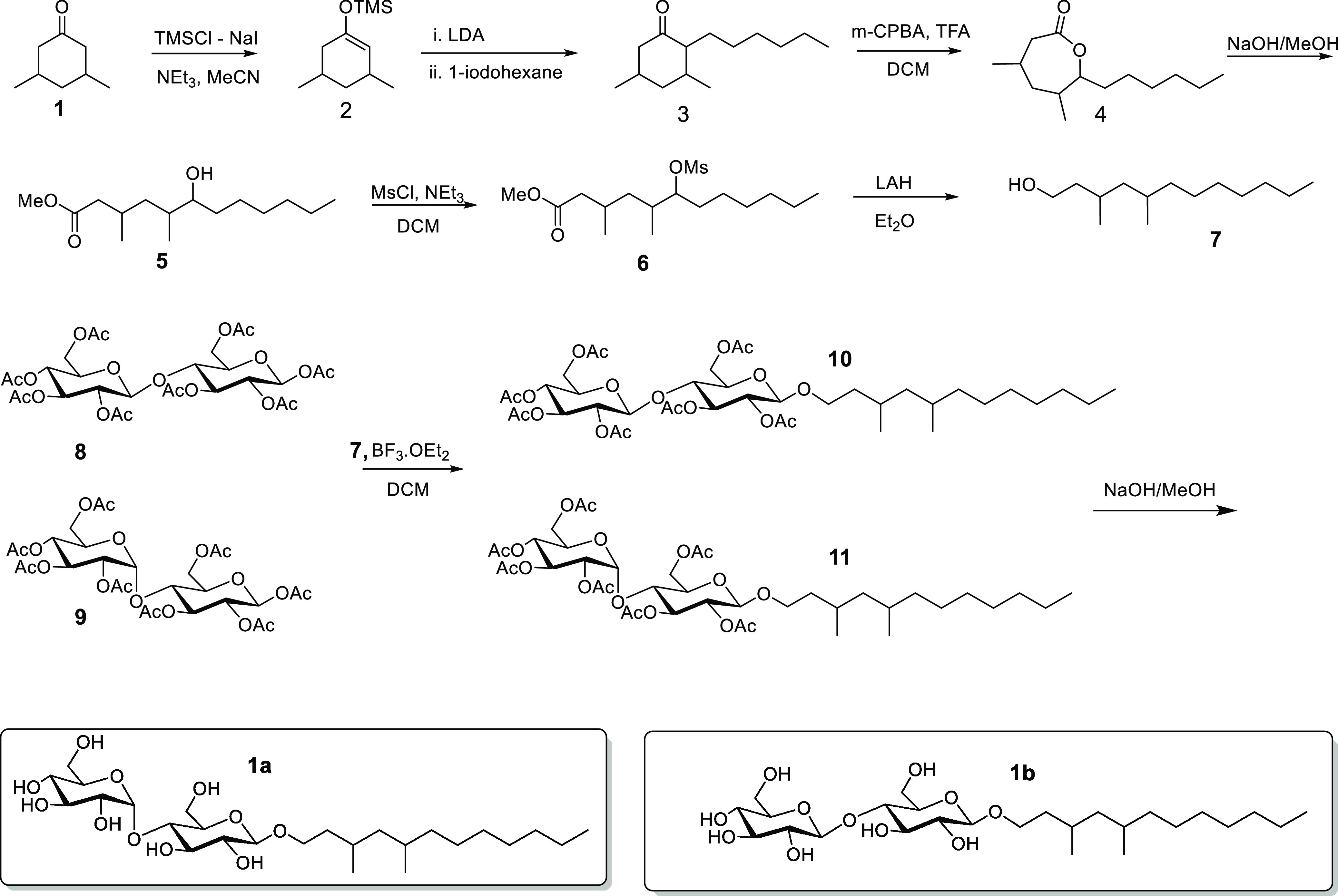
Synthetic scheme for 3,5-diMeDβM (**1a**) and 3,5-diMeDβC (**1b**).

We synthesized the alcohol precursor 3,5-dimethyldodecanol by alkylation of cyclic ketone (3,5-dimethylcyclohexanone), followed by Baeyer–Villiger oxidation. Ring opening was followed by mesylation and one-step ester reduction ester and alcohol mesylation to generate the desired branched alcohol. Glycosylation with protected maltoside and cellobioside followed by basic deprotection generated the final products of 3,5-dimethyl-dodecyl maltoside 3,5-diMeDβM, **1a**, and 3,5-dimethyl dodecyl cellobioside 3,5-diMeDβC, **1b** (Figure 2).

We found that these disugars tethered with the 3,5-dimethyl-substituted dodecyl group exhibited stronger biofilm inhibition activities against *P. aeruginosa* with IC_50_ values of 29 and 36 μM, respectively, whereas SFβM/βC, **2a** and **2b**, from previous studies showed IC_50_ values of 73 and 52 μM, respectively (Table S1). Here, we explore the ability of these molecules to control a wide range of bacterial activities induced by sub-MIC of the antibiotic tobramycin.

### Reversing Phenotypes of *P. aeruginosa* Induced by Tobramycin

In general, use of antibiotics can promote biofilm formation,^3,5^ along with many other resilient phenotypes.^8,17^ A subinhibitory concentration of tobramycin causes a 2-fold increase in biofilm mass formed by wild-type *P. aeruginosa* (*wt* PAO1) on abiotic surfaces^3^ and induces the formation of SCVs in cystic fibrosis patients.^17−19^ It also increases the production of extracellular polymeric substances (EPS)^31^ that also give rise to a rugose/wrinkled colony morphotype when stained with a red dye. More interestingly, tobramycin can also promote the swarming motility of bacteria on hydrated gels.^8^ This particular phenotype development caused by the same antibiotic is intriguing because swarming motility and biofilm formation are inversely regulated by the high and low levels of small-molecule messengers, respectively, in *P. aeruginosa.*(22,23)

**Figure 3:**
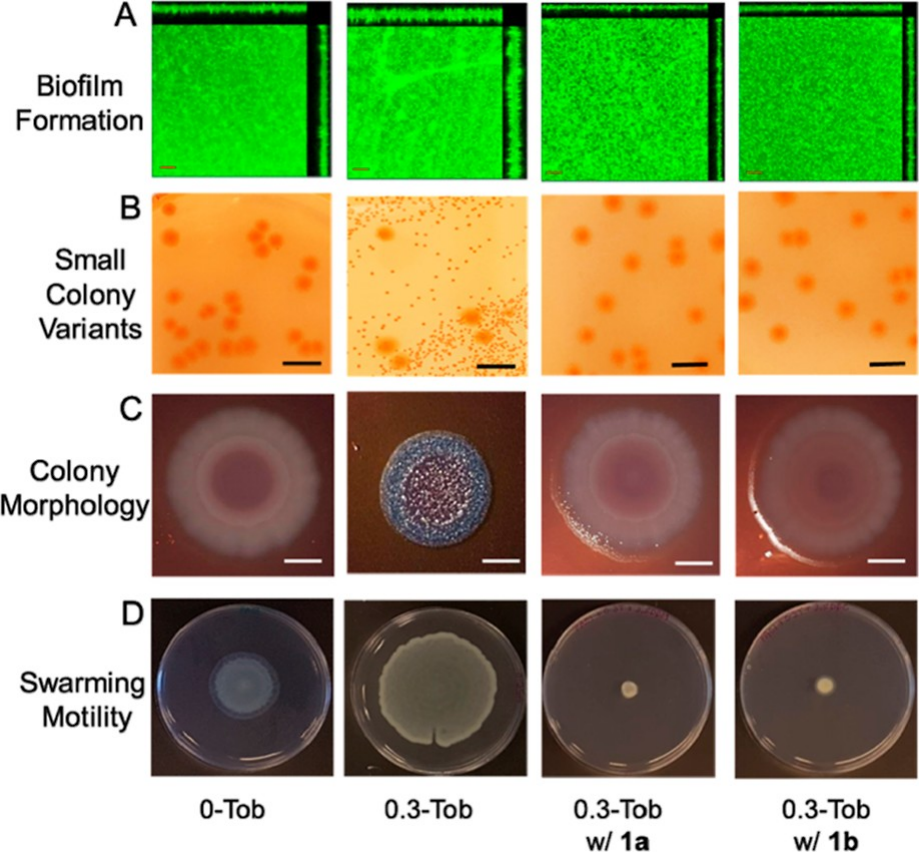
(A) Fluorescent images of unstressed (0-Tob) and 0.3 μg/mL tobramycin (0.3-Tob)-stressed biofilms (24 h) of GFP-tagged PAO1 grown in M63 medium on a polystyrene surface with and without 85 μM **1a** or **1b**. Scale bar = 20 μm. (B) Images of *wt* PAO1 colonies (37 °C, 3 days) on Columbia blood agar plates. Scale bar = 2 cm. (C) Colony morphology of *wt* PAO1 inoculated on 1% agar plates containing Congo red and Coomassie brilliant blue dyes. The colonies were grown for 3 days at 37 °C. Scale bar = 1 cm. (D). Images of swarming patterns of 3 μL of *wt* PAO1 inoculated for 24 h on soft gels (0.5% agar in LB). The agar plates for SCV, colony morphology, and swarming motility contain 0-Tob and 0.3-Tob, with and without 85 μM **1a** or **1b**.

These antibiotic-induced phenotypes all exhibit a high level of tolerance and over time resistance to the antibiotics. While vast research has been carried out for controlling biofilms,^7,20^ it remains unclear whether all these antibiotic-induced phenotypes can be inhibited by a common set of molecules. Here, we found that the small molecules 3,5-diMeDβM and 3,5-diMeDβC, **1a** and **1b**, inhibit all these phenotypes under tobramycin stress. The molecules, **1a** and **1b**, not only inhibited biofilm formation (Table S1) but also reduced 0.3-Tob-stressed biofilm mass by 80% by a crystal violet (CV) staining assay. We validated the inhibition of biofilms using *P. aeruginosa* (PAO1) constituently expressing green fluorescent proteins (Figure S1), with a reduction of biofilm mass by ∼55% (from 27.5 μm^3^/μm^2^ to ∼12.3 μm^3^/μm^2^). This assay demonstrated that 0.3-Tob caused a 40% increase in biofilm mass (from 27.5 to 38.6 μm^3^/μm^2^) on polystyrene surfaces, and when combined with **1a** or **1b** (85 μM), it reduced the biofilm mass by 50% (Figure 3A).

We discovered that subinhibitory amounts of tobramycin also caused SCV formation in vitro (Figure 3B). When *wt* PAO1 were cultured with 0.3-Tob for 6 h, followed by growth on Columbia agar gel containing 0.3-Tob for 24 h, 13-fold of SCVs (1 mm in diameter, 4.2 × 10^7^ CFU/mL) was observed, compared with wild-type colonies (1 cm in diameter, 3.2 × 10^6^ CFU/mL). Under the same condition, combining 85 μM **1a** or **1b** with 0.3-Tob prevented the formation of SCVs entirely (Figure 3B).

To examine the effect of **1a** and **1b** on EPS promoted by tobramycin, the *wt* PAO1 culture (OD_600_ = 0.6) was inoculated on Congo red-containing hard agar plates supplemented with 0.3-Tob for 3 days.^25^ We found that 0.3-Tob caused rugose/wrinkled colony morphotype (diameter, 2 cm) development as revealed by Congo red binding to the exopolysaccharides. In contrast, including 85 μM **1a** or **1b** with 0.3-Tob resulted in a large colony morphotype similar to a normal wild-type morphotype in size (diameter, 3 cm) and smooth texture (Figure 3C). For pellicle formation,^31^ which represents the PEL polysaccharide among the three types of exopolysaccharides, the 0.3-Tob stress increased pellicle formation by 60% at air–liquid interfaces after 3 days without shaking, whereas including **1a** or **1b** (85 μM) completely prevented the ability of 0.3-Tob to promote pellicle formation (Figure S2).

At subinhibitory concentrations, 0.3-Tob in soft gel (0.5 wt % of agar) caused the bacterial swarm area to increase from 24 to 30 cm^2^ (about 25%). When the gel was supplemented with 85 μM **1a** or **1b**, the swarming motility was inhibited entirely in the presence of 0.3-Tob (Figure 3D). Together, these results showed that **1a** and **1b** inhibit both high and low cdG phenotypes that are promoted by tobramycin.

### Inhibition of Tobramycin-Induced Persistent Populations in Biofilms

**Figure 4:**
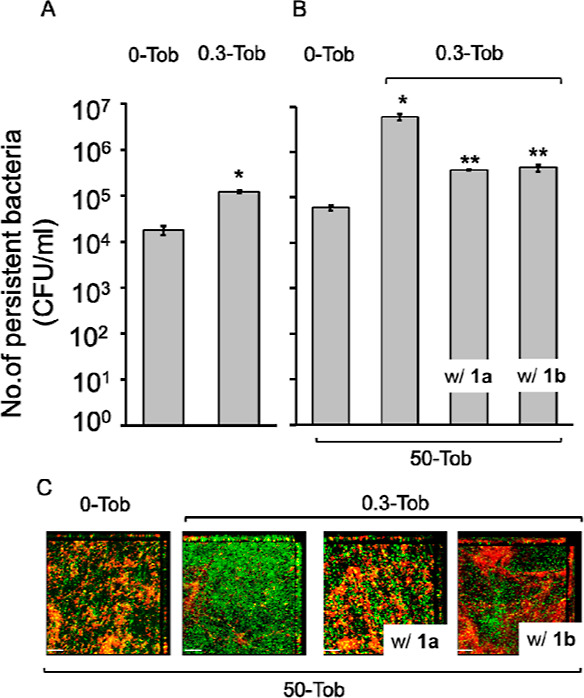
Persistent bacterial count in (A) unstressed (0-Tob) and 0.3-Tob-stressed biofilms (24-h) without 50-Tob treatment. Error bars indicate the standard deviations of means of triplicates. Student’s *t*-test, *P < 0.001 vs 0-Tob biofilms. (B) Unstressed and stressed PAO1 biofilms (24 h) that were further treated with an additional 50-Tob in LB, with and without **1a** and **1b** for another 24 h. Student’s t-test, *P < 0.05; **P < 0.01 vs 0-Tob biofilms. The types of biofilms are shown above the bars, and 50-Tob treatments with and without agents are indicated below. (C) Confocal fluorescent images of unstressed and stressed biofilms of GFP-tagged PAO1 (24 h), further treated with 50-Tob, and with and without 85 μM **1a** or **1b** for another 24 h. The biofilms were stained with propidium iodide dye to show living (green) and dead (yellow/red) bacteria. Scale bar = 20 μm.

High doses of antibiotics are often used to kill bacteria in biofilms, which inevitably lead to antibiotic tolerance and persistence.^1,5^ We first studied the effect of **1a** and **1b** on persistent and tolerant bacteria in unstressed and 0.3-Tob-stressed biofilms. We used a reported method to isolate persistent population.^32^ All biofilms were transferred and sonicated for 15 min in saline to release the bacteria from the biofilms. The saline solution was then treated with 20 μg/mL tobramycin to isolate the persistent population, which was then counted on agar plates without antibiotics. This procedure kills the viable biofilm bacteria, including the tolerant population, resulting in the isolation of the persistent bacteria developed during the drug/agent treatment of biofilm, which revitalize in the absence of antibiotics.^32^ We found that there were about 10 times more persistent bacteria in 0.3-Tob-stressed biofilms (1.2 × 10^5^ CFU/mL) than in unstressed biofilms (1.7 × 10^4^ CFU/mL) (Figure 4A).

To evaluate the effect of high-dose tobramycin on increasing persistent bacteria in a biofilm, 50 μg/mL tobramycin (50-Tob) was applied to bacteria in the already formed biofilms.^32,33^ When unstressed and 0.3-Tob-stressed biofilms were treated with 50-Tob for another 24 h, about 100 times more persistent bacteria were observed in 0.3-Tob-stressed biofilms (∼5.8 × 10^6^ CFU/mL) than in unstressed biofilms (∼5.9 × 10^4^ CFU/mL) (Figure 4B). These results indicate that high doses of tobramycin caused certain precursor bacteria in biofilms to transition to persistent populations. Without 50-Tob treatment, unstressed biofilms increased the persistent population by approximately 3 times, but 0.3-Tob-stressed biofilms increased about 48 times. These results show that stressing biofilms with sub-MIC tobramycin (0.3-Tob) caused a considerable amount of precursor bacteria to form, which can readily transition to persistent populations.

To evaluate the effect of our agents on the persistent population, the molecule **1a** or **1b** (85 μM) was added with 50-Tob. In the presence of either **1a** or **1b**, 50-Tob did not cause an increase of persistent population seen without the agent (Figure 4B), suggesting that **1a** and **1b** prevented the development of a nascent persistent population caused by applying a high dose of tobramycin on biofilms.

To further validate the above results, we grew unstressed and 0.3-Tob-stressed biofilms of GFP-tagged PAO1on polystyrene chips (∼1 cm^2^) and treated the preformed biofilms with 50-Tob for another 24 h and monitored the development of live and dead subpopulations by green and red fluorescence, respectively, by confocal image acquisition. We found that treatment of 50-Tob caused about 8 times more dead bacteria in unstressed biofilms than 0.3-Tob-stressed biofilms. When **1a** or **1b** (85 μM) was added with 50-Tob, there was a substantial decrease in live bacteria and about 10 times increase in dead bacteria in 0.3-Tob-stressed biofilms (Figure 4C).

### Killing Tobramycin-Tolerant Bacteria and Preventing Nascent Persistent Bacteria in Biofilms

**Figure 5:**
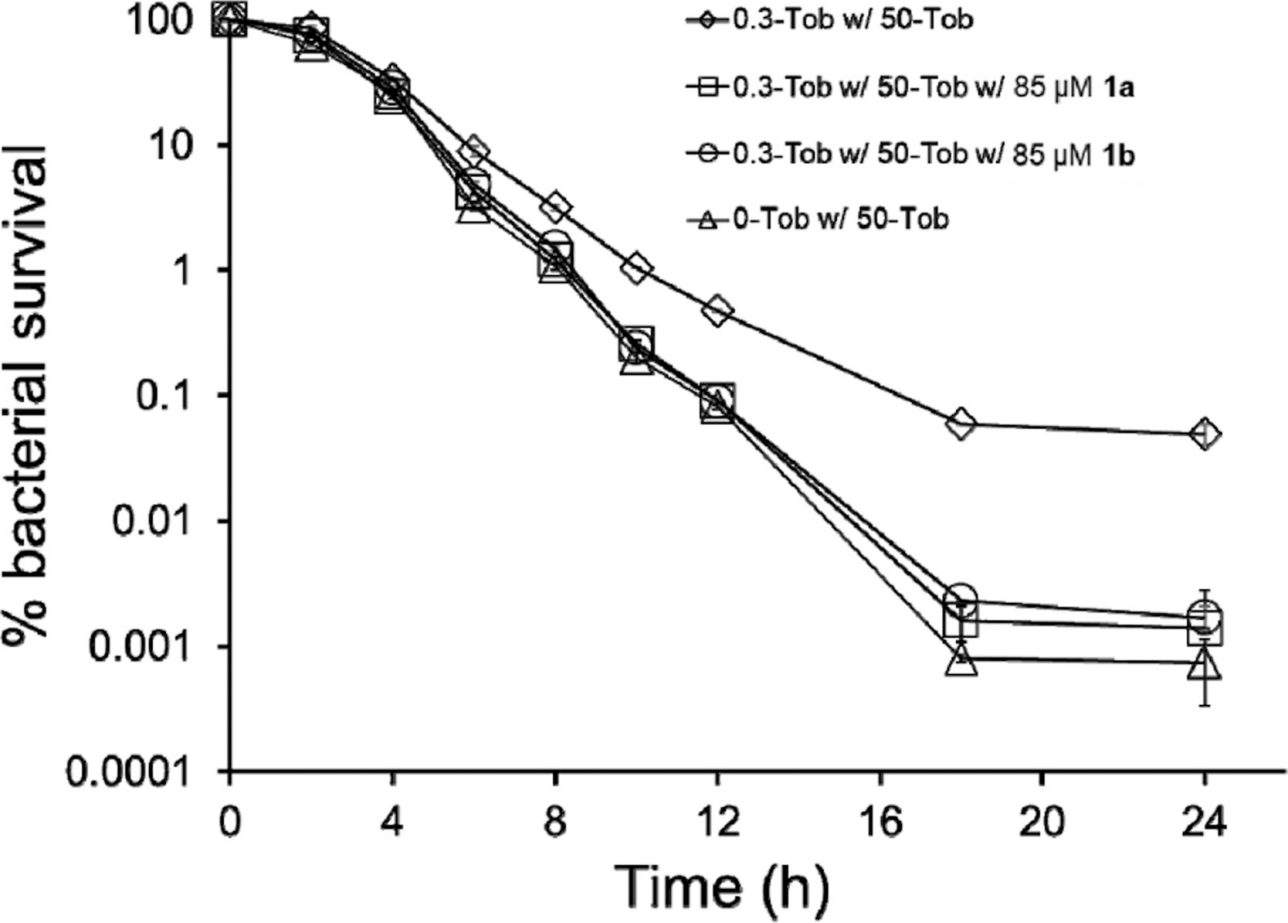
Percentage of bacterial survival (CFU during 50-Tob treatment/initial CFU) of 0.3-Tob-stressed biofilms and unstressed biofilms of *wt* PAO1 (24 h) further treated with 50 μg/mL tobramycin, with and without 85 μM **1a** or **1b**, for different time intervals at 37 °C. Error bars indicate the standard deviations of triplicates.

The kill rate of bacteria revealed the population’s drug tolerance and persistence. We believe the nascent persistent population transitioned from drug-tolerant bacteria.^6,19,34^ To explore this transition and to examine the source of the nascent persistent bacteria in 0.3-Tob-stressed biofilms due to 50-Tob treatment, we performed a time-kill study over 24 h and used the minimum duration for killing 99.9% (MDK_99.9_) of total bacterial population as a measure of bacterial tolerance.^19,34^ Treating the 24 h old biofilms with 50-Tob over another 24 h showed a rapid decrease of viable bacteria for the first 18 h, which plateaued for the remaining 6 h (Figure 5). This biphasic killing curve is consistent with a mixture of susceptible, tolerant, and persistent population. We found MDK_99.9_ for unstressed biofilms to be about 12 h and about 18 h for 0.3-Tob-stressed biofilms (Figure 5). This result indicated that there was a higher population of tolerant bacteria in 0.3-Tob-stressed biofilms than in unstressed biofilms, and the high-dose (50-Tob) treatment converts these tolerant bacteria into persistent populations in biofilms.

Combining 50-Tob with 85 μM **1a** or **1b** reduced the MDK_99.9_ value from ∼18 to ∼12 h (Figure 5). This kill rate is similar to that of unstressed biofilms and suggests that the molecules enabled 50-Tob to kill the pre-existent tolerant bacteria in the 0.3-Tob-stressed biofilms. Between 18 and 24 h, the number of viable bacteria started to plateau for all biofilms. For 0.3-Tob-stressed biofilms, the presence of **1a** or **1b** reduced the bacterial count to ∼3.9 × 10^5^ CFU/mL from ∼5.8 × 10^6^ CFU/mL, further confirming that our molecules prevented the formation of nascent persistent bacteria. Overall, when added to a regiment of high-dose tobramycin, these molecules caused a 100-fold reduction of persistent bacteria in tobramycin-stressed biofilms.

To understand the mode of action, we first confirmed that these molecules do not affect the planktonic growth of *wt* PAO1 (Figure S3), nor are they used as a carbon source by *wt* PAO1 (Figure S4). Because of **1a** and **1b**′s amphiphilic characteristic, we measured their critical aggregation concentrations by a Nile red assay. We found that **1a** self-aggregates at 134 and 140 μM, respectively, in water (Figure S5). All bioactivities against *wt* PAO1 and their mutants are observed at concentrations lower than these aggregation concentrations. Below these aggregation concentrations, we also confirmed that the molecules do not cause hemolysis of human red blood cells (Figure S6). Together with our past study showing that small structural changes have a large impact on inhibition of biofilm formation and swarming motility,^29^ these results are consistent with specific binding of our molecules’ protein receptors, resulting in controlling both high and low cdG phenotypes.

### Identification of Protein Receptors That Control Opposite Signaling Pathways

To identify potential proteins that are the receptors to our molecule, we consider type IV pili and lectin A protein on bacteria. We believe that inhibition of these receptors controls the opposite cdG signaling, leading to different phenotypes. From a structural point of view, both proteins bind sugar moieties,^35,36^ and hydrophobic moieties are reported to be important pili,^35^ and hydrophobic spacers between multivalent sugar molecules are vital for LecA inhibition.^36^ In addition, type IV pili are necessary for swarming and twitching motility;^26,37,38^ and our molecules inhibit both motilities.

For signaling, *P. aeruginosa* utilize type IV pili proteins that transduce environmental cues to signaling events leading to different phenotypes^12−14,38−42^ and facilitate horizontal gene transfer.^43^ In contrast, for biofilm formation, pili appendages initiate surface attachment that eventually triggers the transition into high cdG phenotypes, which lead to antibiotic tolerance.^12,41^ Interestingly, before colonization and biofilm formation, pili-mediated surface sensing increases the quorum sensing that promotes pyocyanin production and induces the type II secretion system.^13^ These virulent phenotypes are the same low cdG phenotype caused by dispersing bacteria from the biofilms.^15^ The SCVs of *P. aeruginosa*, which exhibit high cdG levels, are also hyperpiliated.^18^ Thus, pili appear to mediate transitions between low and high cdG levels and are important for virulence production at both ends of signaling. Inadvertently, type IV pili of *P. aeruginosa* have been shown to assemble over a wide range of cdG levels.^39,44^

Another adhesin LecA, crucial for biofilm formation,^36,45−48^ is positively correlated with the amount of formed biofilms.^46^ The mutant that overproduces LecA forms more biofilms, whereas the mutant of lacking LecA has a low level of cdG and forms a weak/loose biofilm.^46^ Thus, LecA mainly correlates high cdG phenotypes and has been considered as a therapeutic target.^47^ Chemical inhibition of LecA has led to inhibition of biofilm formation.^36,47^

### Direct Ligand–Pilin Interaction Reported by Bacterial Motility-Enabled Binding Assay

As pili are necessary for swarming and twitching^13,26,41^ and bind to different carbohydrate moieties;^35^ we propose that 3,5-diMeDβM/βC, **1a** and **1b**, bind to and inhibit pili appendages. We performed four different experiments to test this hypothesis. First, we found that **1a** and **1b** inhibited not only pili-mediated swarming (Figures 3D and S7) but also pili-mediated twitching motility (Figure S8) and had no effect on swimming motility. These results are consistent with the molecule binding to pili and inhibiting its function.

**Figure 6:**
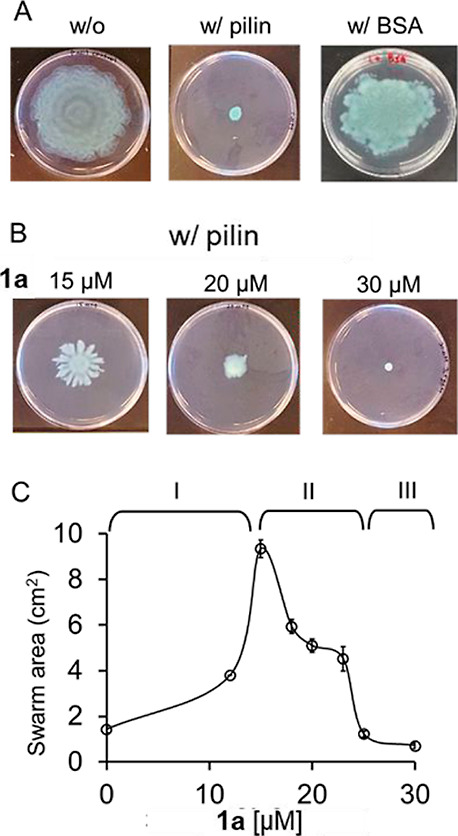
Images of swarming patterns of wt PAO1 (18 h) on the soft gel (0.5% agar in LB) (A) with and without spreading 100 μL of 1 mg/mL pilin or BSA on the gel surface and (B) with the gel containing 15, 20, and 30 μM **1a** and having 100 μL of 1 mg/mL pilin spread on the gel surface. (C) Plot of swarming area of *wt* PAO1 vs concentration of **1a** in the gel, with pilin spread on the surface. Error bars indicate the standard deviations of means of duplicates.

Second, because there are no strong synthetic inhibitors reported for type IV pili, and no well-established binding assays for ligand binding to pili, we created a new binding assay based on modifying the swarming motility experiment that studied the direct binding interactions between pilin proteins and proposed ligand candidates.^49^ We spread a solution of pilin protein on the swarming gel surface and examined if the ligand candidate in the gel will be sequestered by the added protein, depleting the ligands availability on the gel surface. The experimental scheme is that, as pili appendages on bacteria detect the lack of ligand molecules on the gel surfaces, the swarming motility changes. We demonstrated that spreading the pilin proteins that are expressed and purified from a clinical *P. aeruginosa* strain (Figure S9),^50^ PA1244N3-pPAC46 (100 μL of 1 mg/mL, ∼616 nmol), on the gel surface (0.5% soft agar, 10 cm in diameter), inhibited the swarming motility of *wt* PAO1 (Figure 6A). This result is consistent with the hypothesis that the externally introduced pilin proteins on gel surfaces sequester the signaling molecule, likely rhamnolipids secreted by the bacteria,^26,28^ making them unavailable to the pili appendages on the bacterial surface and abolished the bacteria's swarming motility. To test if the expressed pilin proteins bind and sequester our molecules, we spread the pilin protein on swarming gels prepared with different concentrations of **1a** (0–30 μM) and examined the swarming motility of *wt* PAO1 (Figures 6B and S10). We observed that, while pilin alone or just **1a** inhibited the swarming motility, having both pilin on the gel surface and the molecule in gel caused the reactivation of swarming motility of PAO1 over a small range of concentration of **1a**. With 626 nmol of pilin protein (100 μL of 1 mg/mL) spread on a gel surface (10 cm in diameter), PAO1 started to swarm when the concentration of **1a** was increased to 12 μM in gel. The swarming motility reached a maximum (as measured by the area of the swarming pattern) around 15 μM **1a** and decreased as the concentration was increased further (Figure 6C). This reversal of swarming motility as a function of concentration of the molecule is consistent with spread pilin proteins binding and sequestering **1a** from the gel surface. As the concentration of **1a** increases in the gel, binding with pilin proteins abolishes each other’s ability to inhibit the pili appendages on bacteria and thus permitting the swarming motility. When the concentration of **1a** was increased beyond the binding capacity of added pilin proteins, the excess ligand molecules inhibit pili appendages on the bacteria, resulting in reducing the swarming motility again. The peak of around 15 μM **1a** represents the optimal inhibitory concentration against 7.8 nmol/cm^2^ pilin on gel. The control protein bovine serum albumin (BSA) has no effect on *wt* PAO1 swarming.

Third, to directly evaluate the effect of this molecule on the pili appendages of PAO1 surfaces, we examined the proteins sheared from the bacterial surfaces (*wt* PAO1) on agar gel (1.5% agar for growing bacteria),^51^ prepared with 85 μM **1a**. The amount of pilin and other proteins are characterized by using SDS–polyacrylamide gel electrophoresis (SDS–PAGE) with the same protein mass loaded (see the Methods for details). Without the presence of molecules in the hard agar, we observed a band with molecular weight around 16 kD, which is the same as that of the expressed pilin protein from PA1244N3-pPAC46 (Figure S11). For agar gel containing 85 μM **1a**, only a faint band of 16kD was observed, whereas other proteins showed intense bands (Figure S11). This result indicated that the presence of **1a** reduced the amount of pili on bacterial surfaces.

As pili can retract in response to chemotaxis,^12,13^ we examined the effect of our molecules on a nonretracting transposon mutant of pilin assembly protein, pilT(*pilT::Tn*).^52^ We observed that the pilin bands were equally intense for bacteria grown with and without **1a** on agar surfaces (Figure S11). Because our molecules showed no effect on nonretracting pili, we believe that **1a** binding to pili likely caused pilT-mediated retraction.

### Chemical Inhibition of Phage Adsorption on Type IV Pili

**Figure 7:**
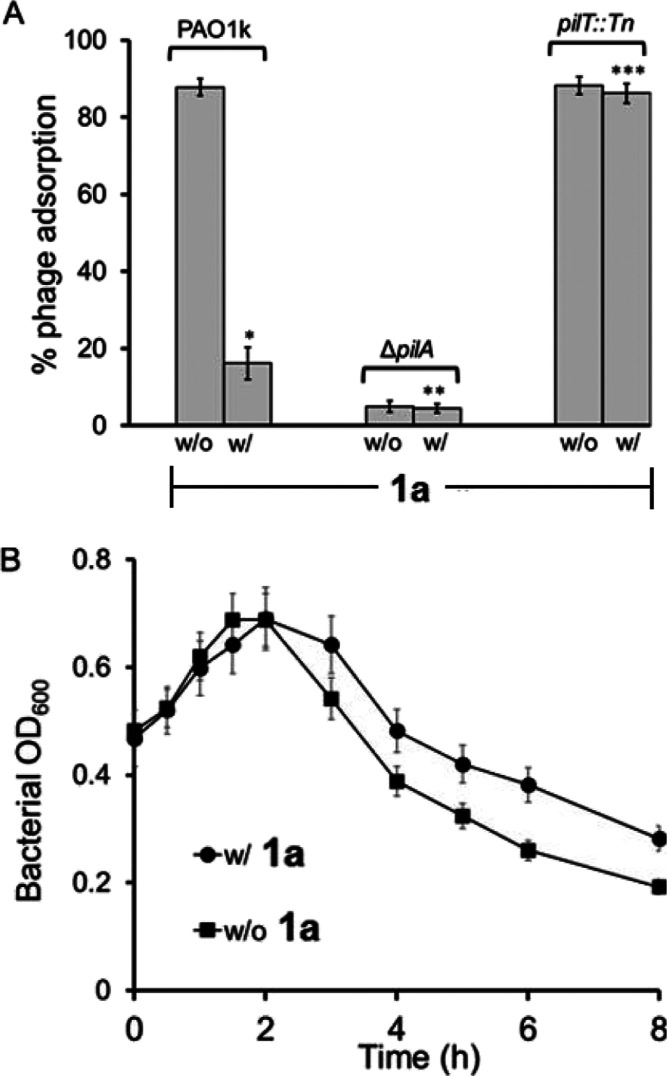
(A) Percentage of ϕKMV phage adsorbed on PAO1k (*wt* PAO1 strain that is sensitive to ϕKMV) and Δ*pilA*, *pilT::Tn* mutants over 10 min in LB containing 85 μM **1a**. Error bars indicate the standard deviations of means of triplicates. *P < 0.01; **P < 0.01 vs control without **1a**, Student’s *t*-test. (B) ϕKMV phage killing rate against PAO1k (OD_600_ = 0.6) cultured with and without 85 μM **1a** for 8 h. The optical density (OD_600_) of the cultures was measured over time. Error bars indicate the standard deviations of means of triplicates.

Last, pili appendages are the key receptor proteins for direct binding and adsorption of bacteriophages, which facilitate the injection of DNA into bacteria. The lytic bacteriophage ϕKMV targets specifically the type IV pili of *P. aeruginosa* for adsorption on pili and subsequent lysis of bacteria.^46,47^ The nonpiliated strain Δ*pilA*^53^ or the retraction mutant *pilT*(*pilT::Tn*) is resistant to lysis by bacteriophages.^52^ Here, we studied the effect of **1a** and **1b** on the bacteriophage ϕKMV adsorption in three *P. aeruginosa* strains: PAO1k, the phage-sensitive strain *wt* PAO1; the pili-deficient mutant Δ*pilA*;^53^ and the nonretracting mutant *pilT*.^52^ We found that by mixing ϕKMV (5.8 × 10^6^ PFU/mL) with excess of PAO1k (2.8 × 10^7^ CFU/mL) for 10 min, the phage titer in the supernatant culture was reduced to 6.8 × 10^5^ PFU/mL, indicating that ∼86% of the phage was adsorbed on PAO1k (Figure 7A). In contrast, upon culturing bacteria in the presence of **1a** (85 μM), the phage count in solution was 4.2 × 10^6^ PFU/mL, indicating only ∼16% of phage adsorption on PAO1k. For the pili-deficient mutant, Δ*pilA*, under the same condition, a low level of adsorption of the added phage (∼4.5%) was observed (Figure 7A). The presence of our agent did not exhibit a noticeable effect on phage adsorption on Δ*pilA*. Because of the lack of pili on Δ*pilA*, we consider this ∼4.5% as a non-pili-mediated phage adsorption. Thus, for PAO1k, the pili-mediated phage adsorption is about 86–4.5 = 81.5% without the agents and about 16–4.5 = 11.5% with the presence of agents. This result suggests that our agents cause about (81.5–11.5)/81.5 = 86% reduction in bacteriophage adsorption on the pili of PAO1.

In the presence of another saturated farnesol derivative SFEG_4_OH, **3** (Figure 1), which inhibits swarming motility, but not biofilm formation,^29^ phage adsorption on pili was also inhibited by ∼72% (Figure S12). To explore the mechanism of the inhibition of phage adsorption, we observed high levels of phage adsorption (∼87%) on the nonretracting mutant *pilT::Tn* (Figure 7A). The presence of **1a** did not exhibit a noticeable effect on phage adsorption on *pilT::Tn*. These results further support that our molecules bind and retract pili appendages of PAO1—likely by the pilT retraction mechanism.^52^

While the pili-deficient mutant Δ*pilA* was resistant (Figure S13), PAO1k was killed by phage ϕKMV in 8 h (Figure 7B). The presence of **1a**, however, caused a delay of killing of PAO1k by 4 h and an increase in residual live bacteria after 8 h (Figure 7B). This result is consistent with the previous finding that the anti-pilin antibody can delay the killing of bacteria by another predator strain through pili binding.^54^ The control molecule SDS has no effect on either phage adsorption or phage-mediated killing. These four different experiments support that our molecules bind to pilin proteins and inhibit the function of pili appendages on bacteria.

### Ligand Binding and Inhibition of LecA

The molecule SFEG_4_OH **3**, a pili ligand, inhibited swarming motility but not biofilm formation. While pili are important, for bacterial adherence and thus for biofilm formation, to understand the reasons for disugar molecules with substituted alkyl chains **1a**, **1b**, **2a**, and **2b** at inhibiting both biofilm formation and swarming motility, we explore another protein, LecA, that is known to bind to sugars^36,45−48,55^ and is important for biofilm formation.^45,47,48^ While LecA of *P. aeruginosa* was initially discovered to have an affinity for galactose, examples of having glucose as part a galactose–glucose disugar have actually been shown to increase its binding to LecA protein as compared to other galactose–galactose disugar.^55^ More notably, the diglucose linked by oligo(ethylene glycol) without any galactose groups exhibited considerably strong binding to LecA.^56^ Varrot and co-workers have demonstrated crystal structures with a secondary sugar binding site for glucose.^55^ It is important to note that galactose and glucose binding to LecA is enhanced by multivalent presentation,^45,47,55,57^ and the space between the binding sites on LecA plays a significant role. Evidently, tethering aromatic groups to single sugar galactose greatly enhance its binding to LecA.^47^ These findings provide an important background for exploring **1a** and **1b** for LecA binding as these molecules consist of glucose units in the form of maltoside and cellobioside.

**Figure 8:**
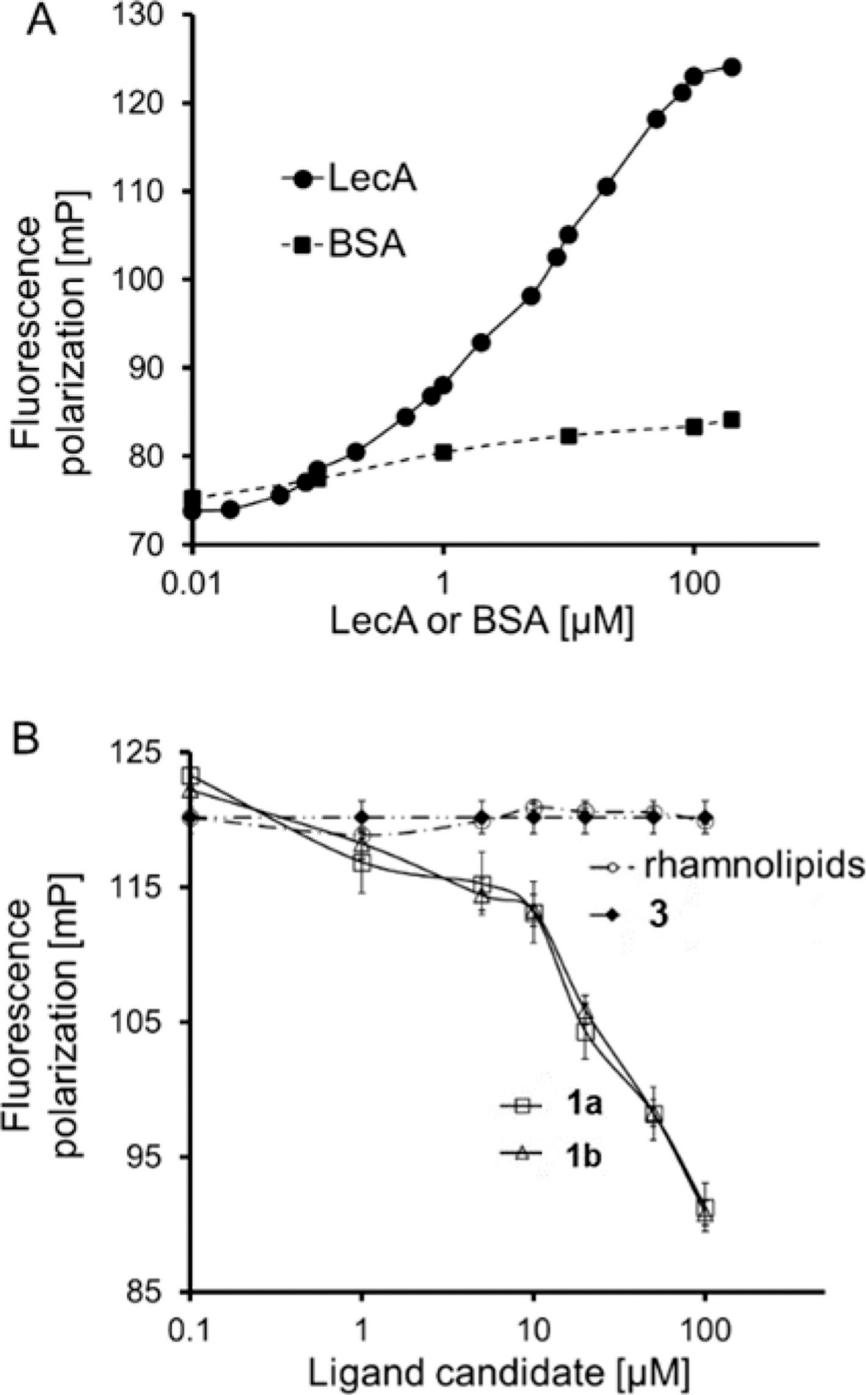
Fluorescence polarization of (A) 200 nM βGal-Dansyl vs concentrations of LecA and BSA proteins and (B) of 100 μL of LecA (20 μM) and βGal-Dansyl (200 nM) vs concentration of candidate ligands, **1a** and **1b**, SFEG_4_OH (**3**), and rhamnolipid mixtures. Error bars indicate the standard deviations of means of triplicates.

We used the fluorescence polarization-based competitive binding assay that has been shown to identify ligands for LecA protein.^58^ For this assay, we designed and synthesized a Dansyl fluorophore-tagged galactose ligand (βGal-aryl-Dansyl, Figure 1) for the LecA protein. The fluorescent polarization of the fluorophore-tagged ligand will increase when bound to a protein receptor because of the reduction in dynamic and will decrease when the fluorophore ligand is displaced from the protein by another ligand. The βGal-aryl-Dansyl (200 nM) showed an increase in its fluorescence polarization with increasing concentrations of LecA (0–100 μM), with a transition indicative of a *K*_d_ ∼10.7 μM, whereas for the control protein, BSA, no significant increase in fluorescence polarization was observed (Figure 8A).

To estimate the binding strength of the potential ligands, **1a** and **1b**, to LecA protein, the molecules were titrated against the complex of βGal-aryl-Dansyl (200 nM) and LecA (20 μM) to displace βGal-aryl-Dansyl from LecA. We found that **1a** and **1b** caused a decrease of fluorescence polarization indicative of displacement of βGal-aryl-Dansyl with an IC_50_ value of 15 and 13 μM, respectively (Figure 8B). Interestingly, rhamnolipids and SFEG_4_OH did not cause a decrease of fluorescence polarization. These results are consistent with the knowledge that glucose-containing disugars are capable of binding to LecA.^55,56^

Binding does not necessarily impact the activities of a protein. Inhibition of biofilms by single galactose derivatives or sugar without attaching groups is often not reported. For our case, β-Gal-Dansyl exhibited a weak biofilm inhibition, IC_50_ 120 μM. Imberty, Romer, and Winssinger and co-workers have shown that the space between the two sugar binding sites has the potential to interact with the group attaching the sugar.^47,57^ We believe that the branch hydrocarbon chain tethering the cellobioside or maltoside plays an enhancing role for binding that space and for controlling the activity of LecA. In this case of having a hydrophobic chain, glucose has an indispensable advantage of having better water solubility over galactose. This binding effect is an ongoing subject of our research.

To further corroborate for these LecA binding studies, we performed a bacterial motility-enabled binding assay.^49^ In this assay, we spread LecA on the soft agar gel containing **1a** that inhibited swarming motility. We observed that while the swarming motility of PAO1 was inhibited completely on gels containing 20 μM **1a**, spreading LecA (100 μL of 1 mg/mL, 780 nmol) on the gel surfaces reactivated the swarming motility (Figure S14). This result is consistent with LecA binding and sequestering **1a** on the gel surface, depleting their availability for pili appendages on bacteria and thus abolishing their swarming inhibition activities. For pilin binding, in contrast, branched aliphatic chains having different water-soluble groups, SFEG_4_OH of **3**, or disugar of **1a** or **1b**, supported binding to pili and inhibition of pili-mediated motilities. These results indicate that **1a** and **1b** are chimeric ligands for LecA and pili, whereas **3** is a ligand for pili alone.

### Chemical Control and Correlation of Receptor Proteins with Their Phenotypes

The chimeric ligands inhibited biofilms—and associated tolerance and persistence, SCVs, rugose colony, and pellicles formation, as well as swarming and twitching motility for wild-type *P. aeruginosa*. Because the two motilities are associated with low cdG levels,^23,39,40^ the other phenotypes are of high cdG levels. We also evaluated other low cdG-controlled virulence factors of *P. aeruginosa*, including the pyocyanin,^24^ proteolytic enzyme-elastase (Figures S15). Pyocyanin production is augmented in low cdG mutants.^24^ Elastase production increases during pili sensing of a surface,^14^ We found that the pili inhibitor alone, SFEG_4_OH, reduced type PAO1’s pyocyanin and elastase levels by 50 and 49%, respectively (Figure S15). This result makes the correlation of inhibiting pilin proteins to inhibit pili-associated activities that also associated with low cdG levels.^39^ The chimeric ligand for both LecA and pili (**1a** or **1b**) also reduced wild-type pyocyanin production by 60% and elastase activity by 45%.

Overall, the chimeric ligands for LecA and pili proteins inhibited all the eight phenotypes (biofilms, SCVs, rugose colonies, pellicles, twitching, swarming motilities, elastase, and pyocyanin production). In contrast, the pili ligand alone, SFEG_4_OH, has no effect on biofilms, SCVs, rugosa, and pellicles but inhibited swarming, twitching motilities, and elastase and pyocyanin production. These results show that the chimeric ligands (for LecA and pili) inhibit the phenotypes for both high and low cdG levels, whereas the pili ligand inhibit only low cdG levels (Table S2). This selective inhibition of LecA and pili enables the correlation between the phenotypes and the receptor proteins. Because inhibiting pili alone by SFEG_4_OH has no effect on biofilms, SCVs and EPS, and pellicle formation, these high cdG phenotypes^45^ are primarily controlled by inhibition of LecA. In contrast, the low cdG phenotypes, overproduction of pyocyanin and elastase, as well as swarming and twitching motilities can be controlled by pili inhibition alone.

Pili retraction resulted from sensing a surface activates virulence factor regulator (*vfr*) gene and type III secretion system, which promotes pyocyanin and elastase, respectively.^12,13^ This important knowledge suggests that pili sensing can feedback to signal to the production of virulence factors correlated with low cdG levels. Our results show that chemical inhibition of pili not only inhibits activities directly controlled by pili (swarming and twitching motility) but also virulence factors (pyocyanin and elastase) that are signaled by pili sensing the environment.

**Figure 9:**
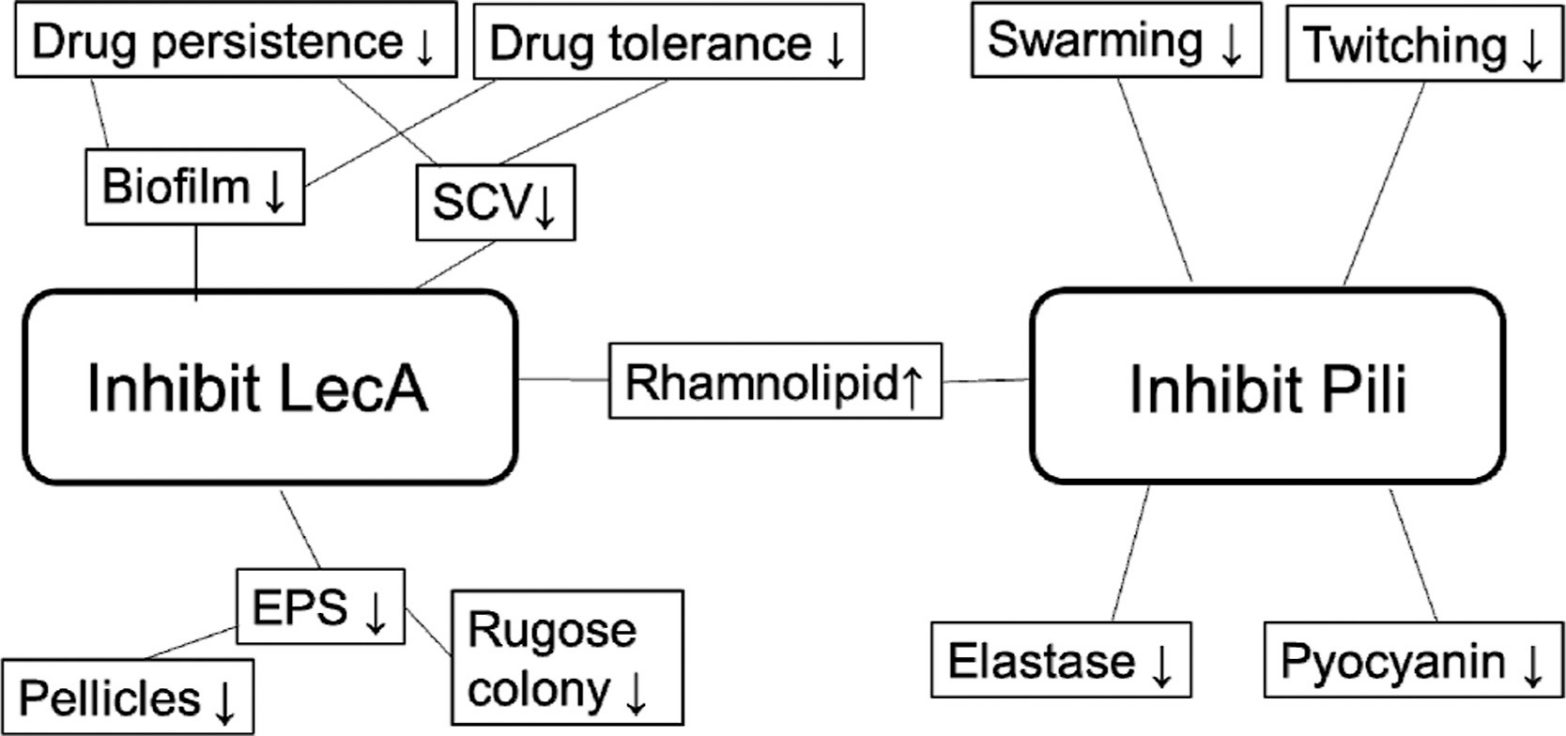
Mapping of chemical inhibition of pili and LecA and the correlation with their controlling phenotypes.

We also evaluated the production of rhamnolipids in the presence of chimeric ligands for LecA and pili and pili ligands alone. Rhamnolipids are a special case in the signaling context because they are needed for a structured biofilm,^27^ which is of high cdG phenotype, but are also necessary for swarming motility,^28^ which is of low cdG phenotype.^24,28^ Interestingly, both SFEG_4_OH and the chimeric ligands cause about a 5-fold increase in rhamnolipid production, to ∼107 μM (Figure S15c), which is close to rhamnolipid’s critical micelle concentration (130 μM).^59^ Because pyocyanin and rhamnolipids are inversely regulated in *P. aeruginosa*,^7^ and our result shows that pili inhibition leads to pyocyanin reduction, it is consistent that pili inhibition also leads to an increase in rhamnolipid production. Furthermore, lowering the cdG level leads to *rhl* quorum sensing that increases rhamnolipid production,^13,15^ and lecA inhibition has been shown to correlate with low cdG;^45^ thus, inhibiting LecA can also promote rhamnolipid production. Together, these results provide a signaling map identifying the phenotypes of pili and LecA proteins and the effects from chemical inhibition of the two proteins (Figure 9 and Table S2). Overall, inhibiting LecA inhibits high cdG phenotypes, whereas inhibiting pili suppresses low cdG phenotypes. We note that pili appendages also relates to drug tolerance as increasing swarming motility is also known to lead to antibiotic tolerance.^9,10^

Compared to a previous set of molecules, **2a** and **2b** (Figure 1) exhibited stronger swarming inhibition but weaker biofilm inhibition than **1a** and **1b**. The molecules **2a** and **2b** also inhibited both high and low cdG activities, including biofilms, SCV and pellicle formation, swarming and twitching motilities, and pyocyanin and elastase A levels, and promoted rhamnolipid production (results not shown). These results indicate that both classes of molecules likely target the same receptors and share a common mode of action. As both pili and LecA are known to recognize and bind sugar moieties modified with derivatives, the results of the molecules **1a**, **1b**, **2a**, and **2b** show the important impacts of the branched aliphatic chains on the two proteins.

It is important to note that there are many signaling pathways and conditions that can lead to biofilm formation and increase in cdG levels, including quorum sensing, starvation, and importantly conventional antibiotic use.^4,60^ LecA is likely not the only protein that controls biofilm formation but our results, and literature,^45,47,48^ suggest that LecA likely is a dominating one.

## Conclusions

In this work, we showed a class of molecules that bind and inhibit both lectin A and pili appendages and another that inhibit pili alone. We showed the selective chemical inhibition of bacteriophage’s adsorption on pili appendages and a label-free assay that identifies direct binding between pilin proteins and chemical ligands. The chemical inhibition of pili appendages likely causes retraction of pili appendages and inhibits phenotypes that are associated with low cdG levels. Chimeric ligands inhibit both LecA protein and pili appendages and phenotypes associated with both high and low levels of cdG. The chimeric ligands also inhibit all the known tobramycin-induced phenotypes, including enabling the killing of tolerant population and the prevention of nascent persistent population in biofilms and the SCVs that are specific to tobramycin stress. Being able to selectively inhibit only pili and inhibit both pili and LecA, these chemical tools delineate that pili appendages are important for low cdG phenotypes, whereas LecA is important for high cdG phenotypes. Overall, these results suggest an adjuvant approach for existing antibiotics by inhibiting the virulent phenotypes and a structural consideration for new drug development. The ligands for pili also present opportunities for inhibiting horizontal gene transfer and related drug resistance. Finally, the label-free bacterial motility-enabled binding assays can be explored for identification and studying new ligand–receptor interactions.

## Methods

Here, we describe experiments in this work that are new or modified from the literature.

### *In Vitro* Small Colony Variant Assay^18^

Overnight cultures (100 μL) of *wt* PAO1 were diluted in 10 mL of LB containing 0.3 μg/mL tobramycin with and without 85 μM **1a** or **1b** and incubated at 37 °C without shaking for 6 h. These cultures were serially diluted (10^5^–10^7^) in LB medium, spread on Columbia blood agar plates (Thermo Scientific Oxoid Columbia Blood Agar Base (Dehydrated) supplemented with 5% sheep blood) containing 85 μM **1a** or **1b**, and incubated at 37 °C for 1 day.

### Bacterial Swarming Assay^28^

The soft gels for swarming motility were prepared by autoclaving 0.5 wt % Bacto Agar in M8 medium (0.6% Na_2_HPO_4_, 0.3% KH_2_PO_4_, and 0.05% NaCl). The gel solution was cooled to ∼60 °C supplemented with filtered 0.2% glucose, 0.5% casamino acid, and 1 mM MgSO_4_ (0.22 μ filter). The gel solutions were poured into a Falcon tube for 20 mL portions, followed by adding aliquots of (1–20 μL) of **1a** or **1b** stock solutions to achieve the desired concentrations. The falcon tubes were closed, and the agar solution was mixed by gently rocking and then poured into polystyrene Petri dishes (10 cm diameter). The agar solution was solidified by cooling and air-drying in a laminar hood for 1 h. The bacterial culture of wild-type PAO1 (3 μL) with an OD_600_ value between ∼0.4 and 0.6 was inoculated on the center of the surface of the soft agar gel. These “swarm plates” were incubated at 37 °C for 12 h and then incubated for an additional 12 h at room temperature. After a total of 24 h, pictures of the swarming plates were taken.

### Bacterial Motility-Enabled Binding Assay

For soft gel prepared for swarming motility (see above), 100 μL of 1 mg/mL pilin, LecA, or BSA protein was introduced onto the center of the agar gel and immediately spread over the surface of the gel using a sterile cell spreader. The proteins were prepared in NaPB (4 mM, pH 7.2), Tris–HCl (0.1 M Tris–HCl, pH 7.5, and 6 μM CaCl_2_), and Tris buffer (2 mM Tris, 7 mM NaCl, and pH 7.5). The agar gel spread with the protein solution was dried for an additional 30 min. A bacterial subculture (3 μL, 0.6 OD_600_) was inoculated on the center of the soft gel (10 cm diameter plate). The bacteria on the soft gel were incubated for 12 h at 37 °C and for an additional 12 h at room temperature.

### Persistent Population Isolation Assay^32,33^

An overnight culture (100 μL) of bacteria in LB was diluted in 10 mL of M63 and incubated to reach an OD_600_ value of ∼0.1. Aliquots (150 μL) of the subculture were added into the wells of a 96-well MBEC microtiter plate with and without 0.3 μg/mL tobramycin, 85 μM **1a** or **1b**. MBEC pegs were immersed in the bacterial culture and further incubated at 37 °C without shaking for 24 h to form biofilms. The MBEC peg lid was then removed, and pegs were washed with 0.9% saline to remove unattached or loosely attached bacteria and antibiotics. The biofilm mass was quantified by CV dye assay for six pegs. The 10 unstained pegs were cut with sterile pliers and suspended in saline water. The saline solution with pegs was sonicated for 15 min at 30 kHz (Symphony VWR Internationals Ltd.). Pegs were removed, and the solutions were centrifuged at 6000 rpm for 10 min to collect the bacteria. The collected pellets were resuspended and supplemented with 20 μg/mL tobramycin (20× MIC) in MHB medium and incubated at 37 °C with shaking at 250 rpm for 8 h to isolate persistent population. The sample was then centrifuged at 8000 rpm for 10 min at room temperature to collect the persister pellet. The supernatant was discarded, and the bacterial pellet was washed twice with 0.9% saline. The number of persistent bacteria was then quantified by counting CFU on an MHB agar plate.

### Chemical Inhibition of Bacteriophage Adsorption ϕKMV on *P. aeruginosa* Strains^52,53^

The bacteriophage adsorption assay was adopted as described previously,^53^ with the addition of our agents. An overnight culture (100 μL) of *wtP. aeruginosa* strain PAO1k that is ϕKMV sensitive, knockout mutant Δ*pilA*, and *transposon* mutant *pilT* (*pilT::Tn*) were diluted with 10 mL of LB supplemented with 10 mM MgSO_4_ (LB-Mg^2+^) and subcultured to an OD_600_ value of around 0.6 at 37 °C with shaking at 250 rpm with and without 85 μM **1a** or **1b** or 60 μM SFEG_4_OH. The bacteria subculture (100 μL) was mixed with 900 μL of LB-Mg^2+^/mL ϕKMV phage. The titer of the added phage was individually determined for every experiment from the phage stock solution. Following incubation for 10 min at 37 °C with shaking at 100 rpm, bacteria were removed by centrifugation (10,000*g*, 5 min at 4 °C), and 900 μL of the supernatant was transferred to an Eppendorf tube. The plaque-forming units (PFU) in the supernatant with and without the added agents were determined by the top agar overlay method with PAO1k. The percentage of phage bound to bacteria was calculated as [(titer of the added phage – titer in the supernatant)/(titer of the added phage)] × 100.

## Supporting Information Available

Synthesis details and ^1^H and ^13^C NMR spectra and mass spectroscopy data of intermediates and final compounds and experimental details for biological assays, bacterial strains, and phage (PDF)
