# Supplementary material for: Chimeric Ligands of Pili and Lectin A Inhibit Tolerance, Persistence, and Virulence Factors of Pseudomonas aeruginosa over a Wide Range of Phenotypes
Source: ACS Infect Dis. 2022 Jun 6;8(8):1582–93. doi: 10.1021/acsinfecdis.2c00201 (PMC9379910; doi:10.1021/acsinfecdis.2c00201)
Supplement: Supplementary file 1 — id2c00201_si_001.pdf [file id2c00201_si_001.pdf]

Supporting Information

Chimeric Ligands of Pili and Lectin A Inhibit Tolerance, Persistence, and Virulence Factors of *Pseudomonas aeruginosa* over A Wide Range of Phenotypes

Pankaj D. Patil, Hewen Zheng, Felicia Burns, Arizza Chiara S. Ibanez, Yuchen Jin, and Yan-Yeung Luk*

Department of Chemistry, Syracuse University, 1-014 Center of Science and Technology, Syracuse, NY, 13244-4100, USA.

*Corresponding author email: yluk@syr.edu

Table of Contents

1. **Supporting figures (Figure S1-S15)**
2. **Materials and Methods**
3. **Synthesis and spectral data (Figure S16-S30)**
4. **References**

1. Supporting Figures

Table S1. 3,5-diMeDβM/βC are more potent than SFβM/βC at inhibition and dispersion of wild type *P. aeruginosa* (*wt* PAO1) biofilms.[a]

Molecule	Biofilm Inhibition IC_{50} ± s.d. [μM][b]		Biofilm Dispersion DC_{50} ± s.d. [μM][c]	
	w/o Tob	w/ 0.3 μg/ml Tob	w/o Tob	w/o 0.3 μg/ml Tob
SFβM	73 ±2 μM*	85±4.6 μM	126±5.5 μM*	>150 μM
SFβC	52±2.4 μM*	79±3.3 μM	83±3.3 μM*	>150 μM
3,5-diMeDβM	36±2.2 μM	62±2.2 μM	54±5.5 μM	98±3.3 μM
3,5-diMeDβC	29±3.2 μM	58±2.1 μM	48±4.6 μM	95±2.9 μM

a. The biofilms were characterized by a Crystal violet staining assays on the pegs of MBEC™ microtiter plates **b.** IC_{50} values were obtained by plotting concentration of the molecule against % biofilm inhibition after 24 h without shaking at 50% of biofilm inhibition. **c.** DC_{50} values were obtained by plotting concentration of molecule against % biofilm dispersion after 24 h with shaking at 100 rpm shaking, at 50 % biofilm dispersion. Experiments were performed in triplicate and the results are an averaged value; error bars reflect the standard deviation of each point. The curve fitting was performed with GraphPad Prism 6.0 software.*indicated values extracted from literature[1]

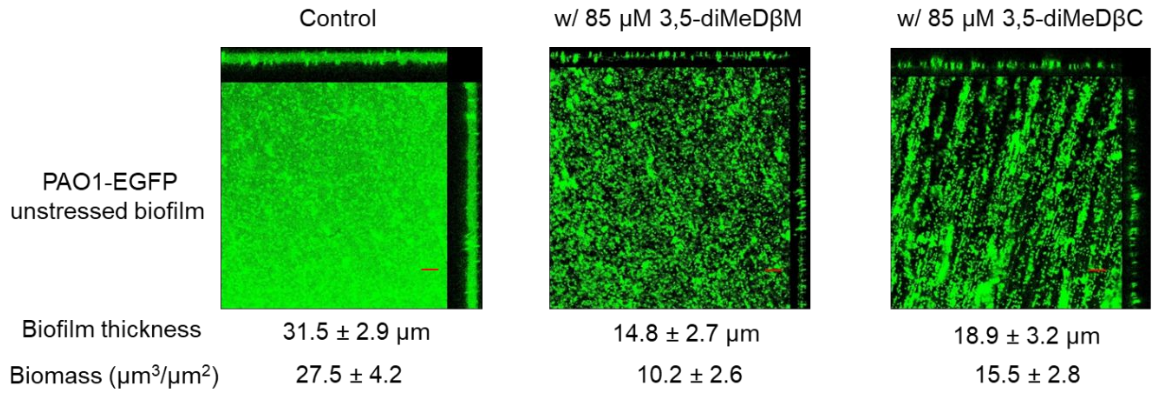

Figure S1. 3,5-diMeDβM/βC inhibit unstressed biofilm formation in *P. aeruginosa*. 24 h biofilms of GFP-tagged PAO1 were grown in M63 medium with and without 85 μM 3,5-diMeDβM/βC on polystyrene surface. Images were acquired by confocal laser scanning microscope. The central pictures show horizontal optical sections, and the flanking pictures show vertical optical sections. *P. aeruginosa* biomass was quantified with the COMSTAT program. Scale bars, 20 μm. Emission of 488 nm was used to visualize live cells within the biofilms formed by PAO1-EGFP strain. The biomasses fluorescing green reveal the amounts of living bacteria in a biofilm. The COMSTAT program was used to report the percentage of live biomasses in the stacks of images.

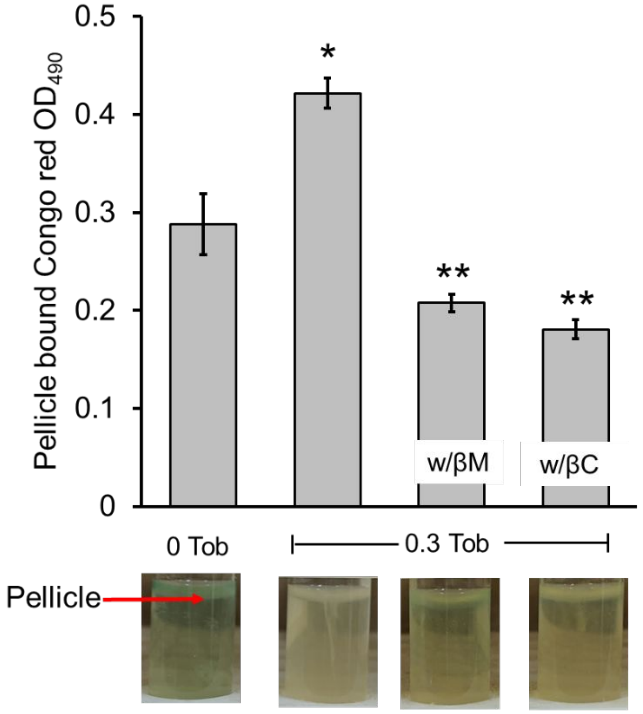

Figure S2. 3,5-diMeDβM/C inhibit 0.3-Tob induced pellicles in static wild type *P. aeruginosa* cultures. Pellicle formation in *wt* PAO1 were observed and quantified by Congo red (CR) binding assay. Pellicles were formed for 3 days under static conditions. Overnight *wt* PAO1 bacterial cultures were diluted 1:100 in LB medium without NaCl with and without 85 µM 3,5-diMeDβM (βM) or 3,5-diMeDβC (βC) in the presence of 0.3 µg/ml tobramycin (0.3-Tob). The cultures were grown statically (in glass tubes) for 3 days at 37°C. Then Congo red binding by pellicles was calculated as the difference between absorbance for reference solution containing the starting concentration of Congo red (40 µg/ml) and the absorbance of the supernatant. Error bars indicate standard deviations of independent triplicates. The pellicles formed without tobramycin (0-Tob) was considered as control. *P = 0.02 vs control; **P <0.005 vs control as evaluated using two-tailed unpaired Student's *t*-test

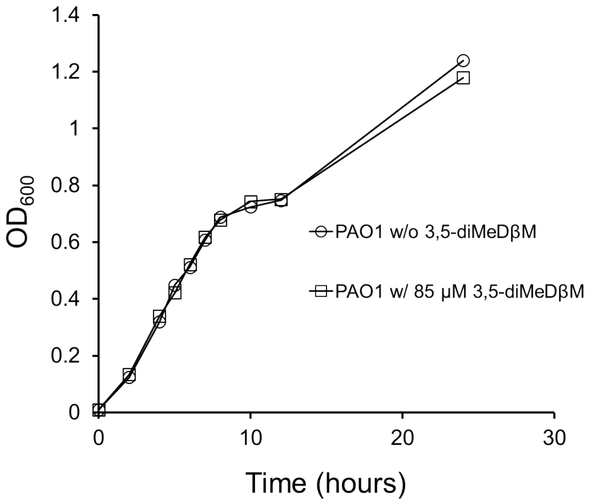

Figure S3. 3,5-diMeDβM does not affect growth of wild type *P. aeruginosa*. The diluted overnight *wt* PAO1 culture (1:100) in MHB with and without 85 μM 3,5-diMeDβM were incubated for 24 h under 250 rpm shaking conditions and optical density OD_{600} was observed during 24 h .

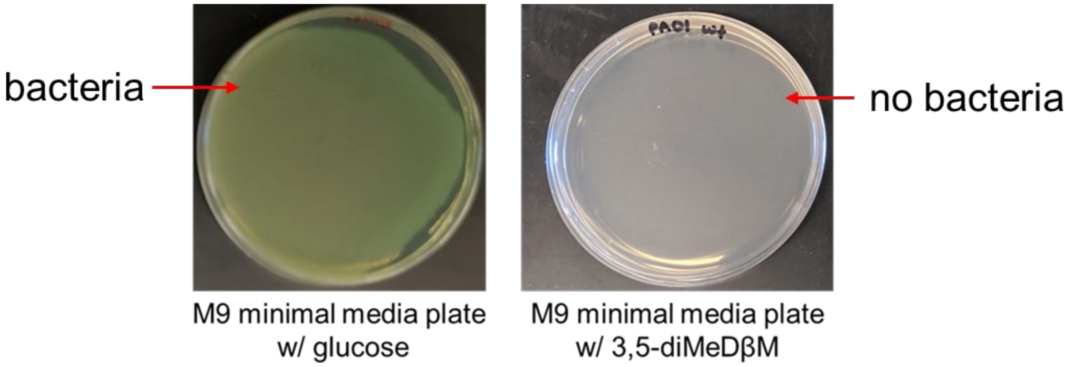

Figure S4. Wild type *P. aeruginosa* does not consume 3,5-diMeDβM as a carbon source. The 100 μL of overnight culture of *wt* PAO1(OD_{600} = 1.0) was spread on M9 minimal media plate with glucose or 3,5-diMeDβM as a sole carbon source. The bacterial growth was observed over 48 h.

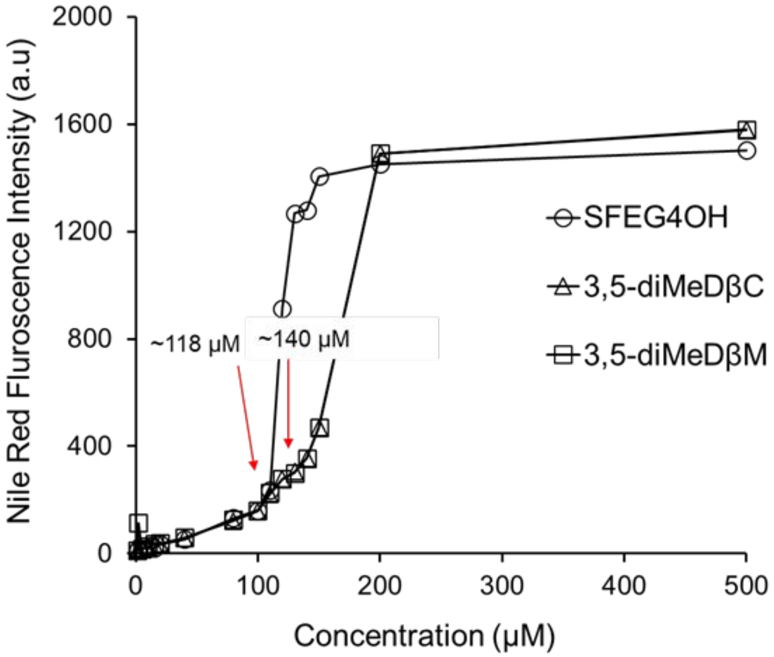

Figure S5. The critical aggregation concentrations for 3,5-diMeDβC, 3,5-diMeDβM, and SFEG$_4$OH was ~140 µM, 138 µM and 118µM. Nile red fluorescence intensity against concentration of 3,5-diMeDβC, 3,5-diMeDβM, and SFEG$_4$OH in deionized water. The critical aggregation concentrations (CAC) correspond to the concentration at the transition of increase of Nile red fluorescence.

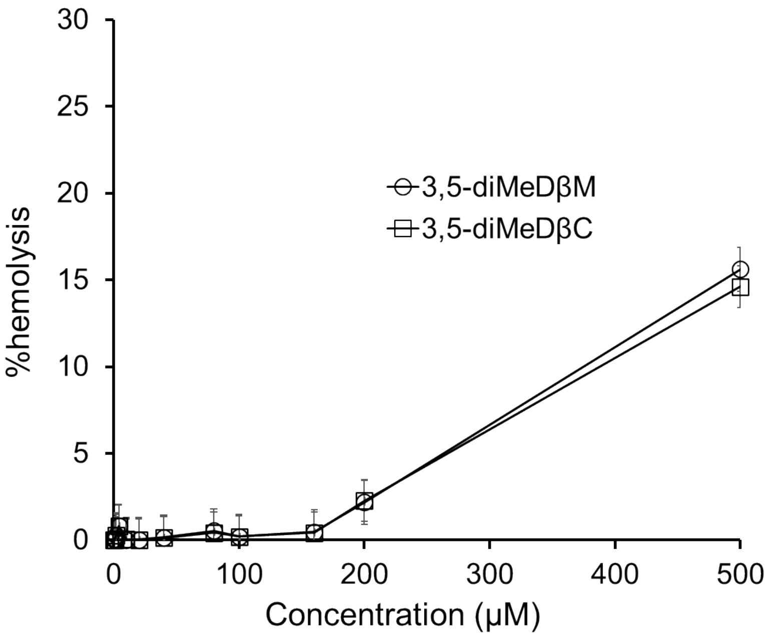

Figure S6. 3,5-diMeDβM/βC are not hemolytic against human red blood cells. The human red blood suspension was incubated with different concentrations of 3,5-diMeDβM/βC for 1 h and the release of hemoglobin was monitored by measuring the absorbance of the supernatant at 550 nm. The % hemolysis was recorded as % hemolysis = $(OD_{(\text{test molecule})} - OD_{(\text{PBS control})})/(OD_{(\text{Tween 80})} - OD_{(\text{PBS control})}) \times 100\%$.

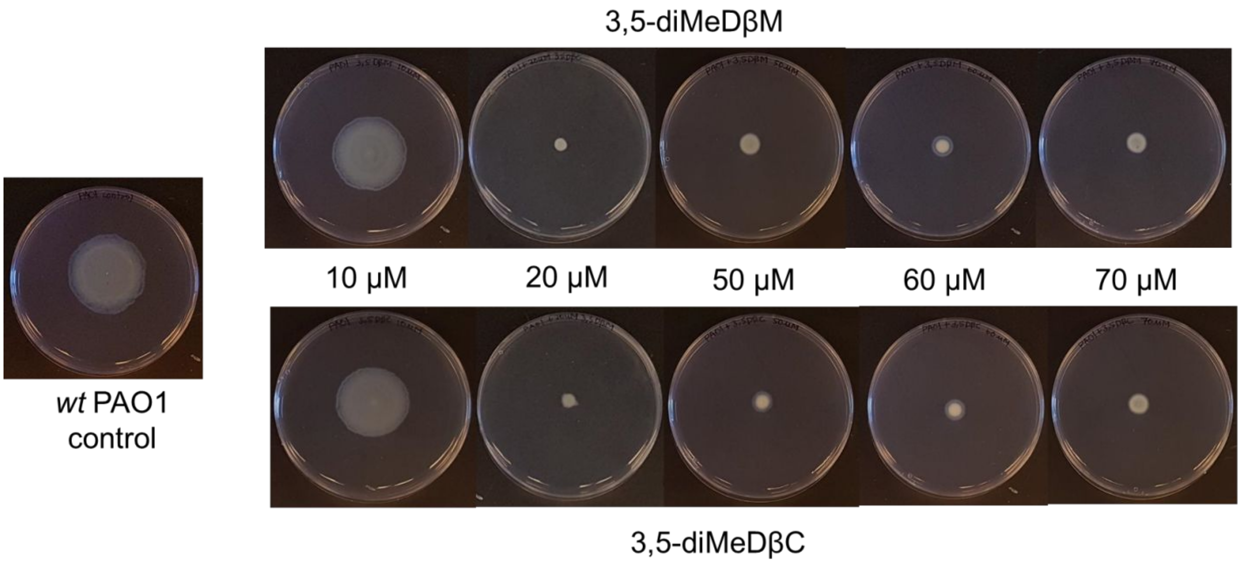

Figure S7. 3,5-diMeDβM/βC inhibit swarming motility of wild type *P. aeruginosa* Swarming motility assay; The representative images of swarming motilities *wt* PAO1 on semisolid gel (~0.5% agar) with different concentrations of 3,5-DiMeDβM/βC after 24 h. The concentrations are indicated between the images.

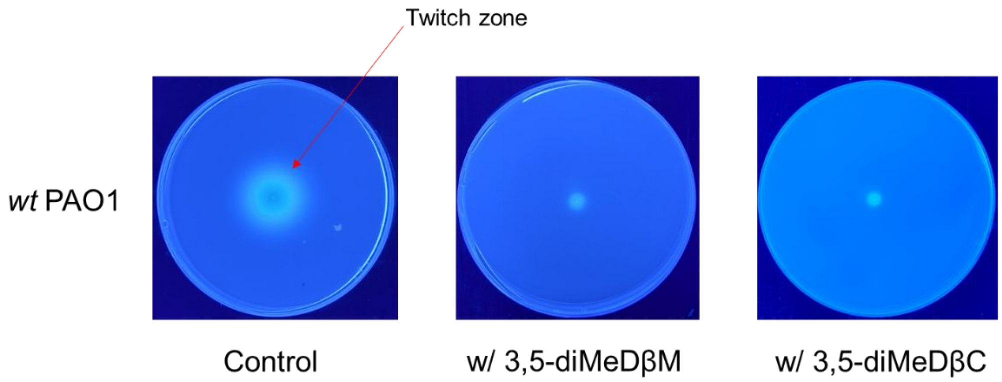

Figure S8. 3,5-diMeDβM/βC inhibit twitching motility in wild type *P. aeruginosa*. 3,5-DiMeDβM/βC inhibit pili-mediated twitching motility in *wt P. aeruginosa*. LB agar plates (1%) were stab inoculated with a toothpick to the bottom of the plate and incubated for 48 h at 37 ° C with and without 30 μM 3,5-diMeDβM/βC. The light haze of growth, the twitch zone, at the agar-plate interface is a measure of twitching motility (red arrow). The smaller, denser zone represents surface colony growth. Images were taken under 365/254nm UV irradiation.

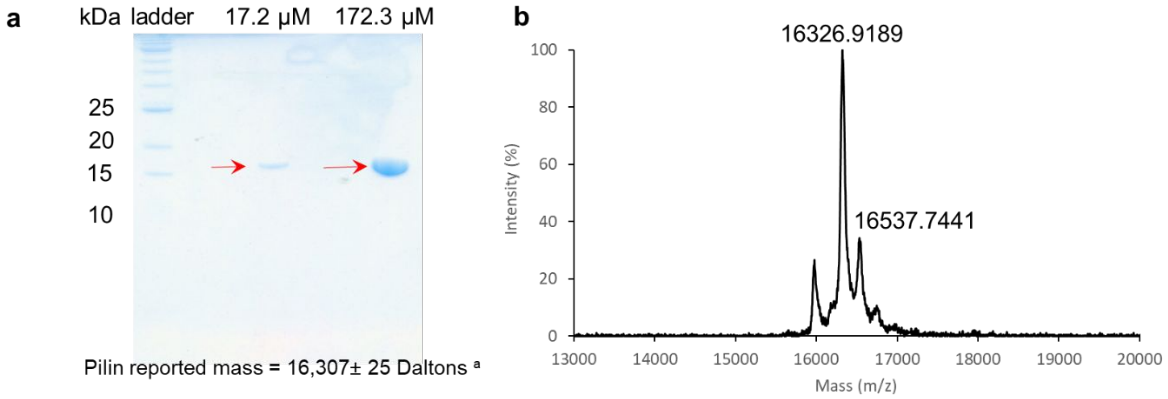

Figure S9. Expressed and purified pilin from clinical *P. aeruginosa strain*, PA1244N3 (pPAC46) showed molecule weight around (a) 16 kD by SDS-PAGE and (b) Molecular weight of pilin 16,326±25 Daltons by MALDI-MS[2-3]. The pilin protein was expressed from a clinical *P. aeruginosa strain*, PA1244N3 (pPAC46), a clinical isolate from burn wound patients, that has an inactive *rpoN* gene resulting in the deficiency in pili protein expression[2-3].

Round I

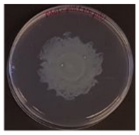

d 5.6 cm
Area 24.63 cm^2

PAO1 Control

PAO1 + 1 mg/ml Pili

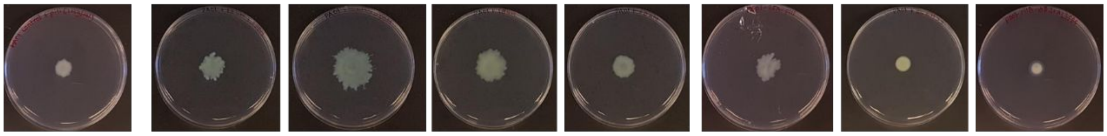

3,5-diMeDβM	0 μM	12 μM	15 μM	18 μM	20 μM	23 μM	25 μM	30 μM
d in cm	1.4	2.2	3.4	2.8	2.6	2.5	1.3	0.9
Area in cm^2	1.54	3.8	9.07	6.15	5.3	4.9	1.32	0.63

100 µL of 1 mg/ml pili protein was spread on agar surface before bacterial inoculation

Round II

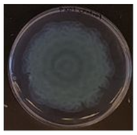

d 5.8 cm
Area 26.42 cm^2

PAO1 Control

PAO1 + 1 mg/ml Pili

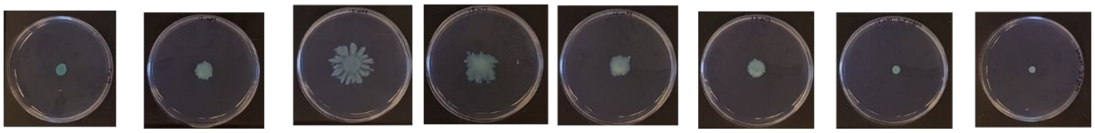

3,5-diMeDβM	0 μM	12 μM	15 μM	18 μM	20 μM	23 μM	25 μM	30 μM
d in cm	1.3	2.2	3.5	2.7	2.5	2.3	1.2	1.0
Area in cm^2	1.32	3.8	9.62	5.72	4.9	4.15	1.13	0.78

100 µL of 1 mg/ml pili protein was spread on agar surface before bacterial inoculation

Figure S10. Externally added pilin protein and 3,5-diMeDβM in gel neutralize each other's swarming inhibition activities. Repetitions of pili-ligand binding assay. 100 µL of pili protein (1mg/ml) expressed from (PA1244N3-pAC46) was spread on the surface of 0.5% agar (10 cm in diameter) containing different concentrations (0-30 µM) of 3,5-diMeDβM, followed by bacterial inoculation (3 µL of culture, OD_{600} = 0.6). Pictures were taken after the plates were incubated at 37 ℃ for 12 h and then room temperature for another 12 h. The swarming area was estimated by measuring diameter of swarm area on agar gel.

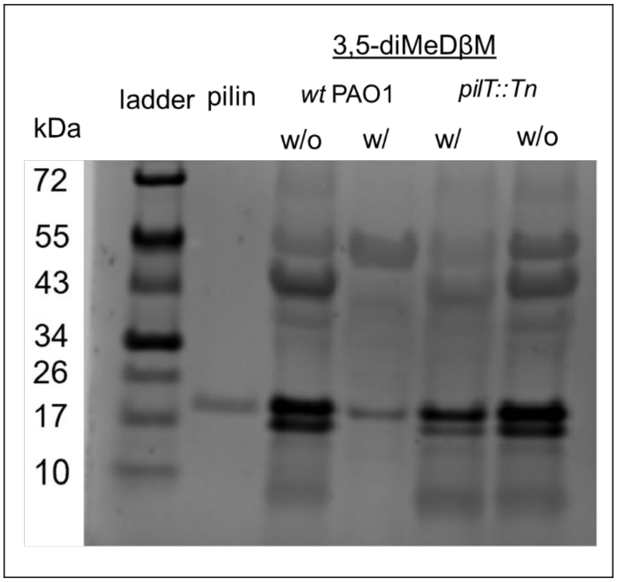

Figure S11. ***P. aeruginosa*** **treated with 3,5-diMeDβM have less surface pili.** Gel electrophoresis (SDS-PAGE) of sheared surface proteins of *wt* PAO1, *pilT::Tn* grown on 1.5% agar with and without 85 µM of 3,5-diMeDβM. From left to right lanes: molecular size markers, standard of expressed pilin from PA1244N3-pAC46, proteins from PAO1 and *pilT::Tn* without and with 3,5-diMeDβM.

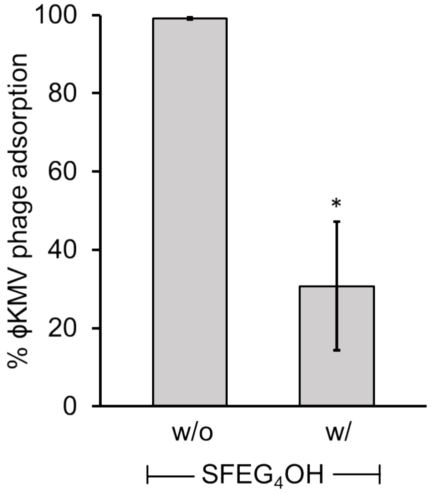

Figure S12. SFEG$_4$OH inhibits bacteriophage adsorption on *P. aeruginosa* PAO1k with functional pili. PAO1k (6x10^7 CFU/ml) pretreated with and without 60 µM SFEG$_4$OH and infected with ~10^6 PFU/ml phage, 10 min, 100 rpm. The phages left in supernatant (not adsorbed on pili) were quantified by plaque formation assay. The % phage adsorption was calculated as 1-(titer in supernatant/ titer of phage added) ×100.[4] Error bars indicate standard deviations of independent triplicates. *P=0.01 vs control as evaluated using two-tailed unpaired Student's *t*-test.

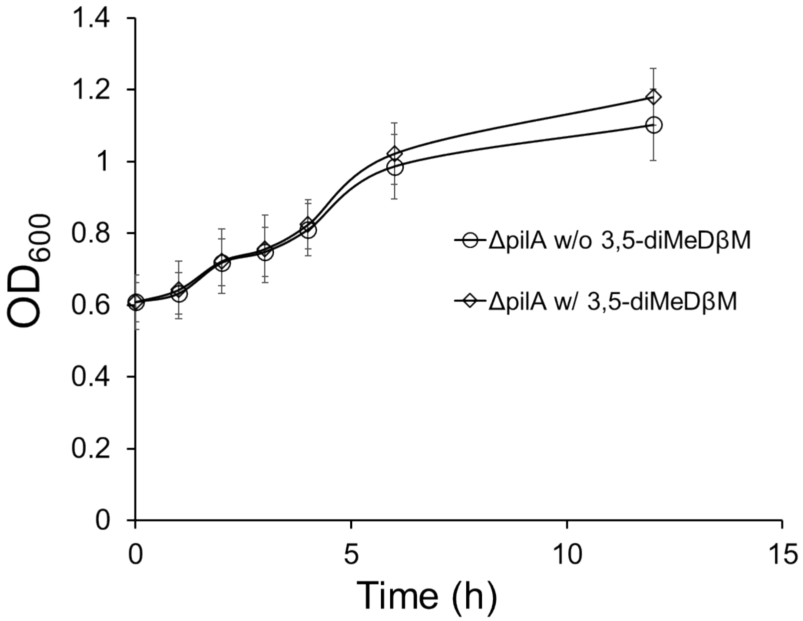

Figure S13. ϕKMV phage with and without 3,5-diMeDβM does not kill pili-deficient mutant (Δ*pilA*). Overnight cultures of Δ*pilA* were diluted 100-fold, and subcultured with the presence of 10 mM $MgSO_4$, and with and without 3,5-diMeDβM (85 µM), to an OD_{600} of 0.6, and then infected with ϕKMV phage (PFU/ml ~10^8). The optical density (OD_{600}) of the bacterial cultures were measured after 12 h. Cultures were incubated at 37℃ at 250 rpm.

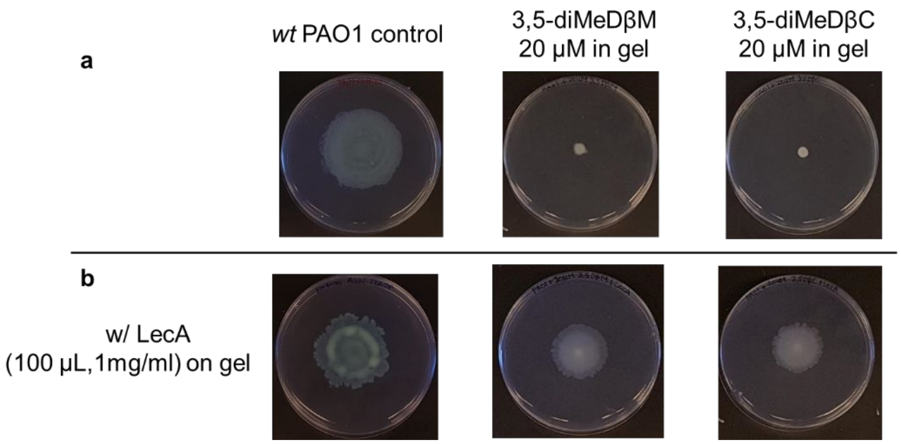

Figure S14. LecA remove effect of 3,5-diMeDβM/C on swarming motility. Swarming of *wt* PAO1 on 0.5% agar (10 cm in diameter) containing 20 µM of 3,5-diMeDβM/βC without **(a)** and with **(b)** 100 µL of LecA protein (1 mg/mL) was spread on the agar gel surface. Upon inoculation, the plates were incubated at 37 ℃ for 12 h and then at room temperature for another 12 h, followed by taking the pictures.

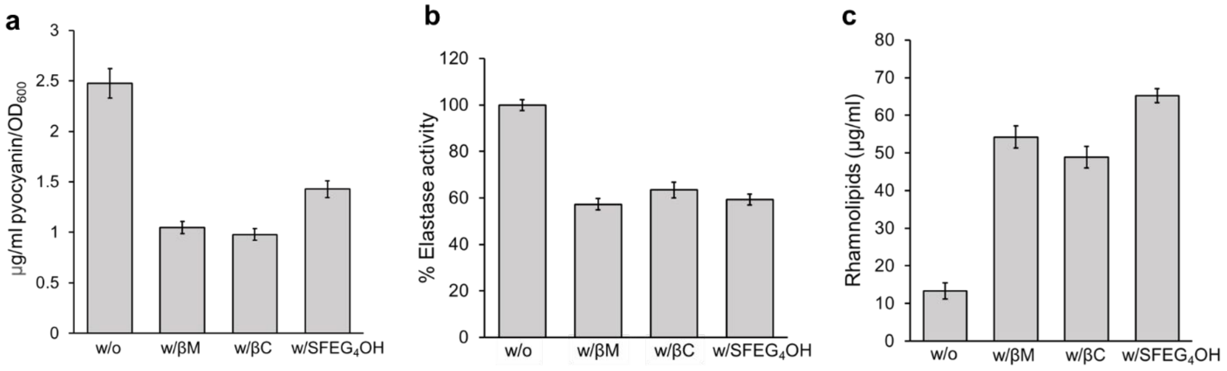

Figure S15. ***P. aeruginosa*** **Virulence assay. (a**) Pyocyanin levels in supernatant of planktonic cultures of *wt* PAO1 treated with and without 85 μM of 3,5-diMeDβM/βC, $SFEG_4OH$ for 12 h in LB. The pyocyanin levels were measured as μg/ml/OD_{600} of the bacterial culture. (**b**) Elastase production in the planktonic culture of *wt* PAO1 treated with and without 85 μM of 3,5-diMeDβM/βC, $SFEG_4OH$ for 18 h were determined using the Elastin-Congo Red (ECR) assays. The % of elastase activity was calculated as OD_{495} (with agent) - OD_{495} (without agent). **(c)** Rhamnolipids productions by planktonic cultures of *wt* PAO1 treated with and without 85 μM of 3,5-diMeDβM/βC, $SFEG_4OH$ for 18 h were determined using an established methylene blue complexation assays.[25] The positively charged methylene blue form noncovalent complex preferentially with negatively charged surfactants and does not bind to nonionic surfactants. The methylene blue bound with negatively charged surfactants has an increased partition into the organic chloroform phase bought in contact with the aqueous solution. The methylene blue in the chloroform phase were measured for its UV absorbance, which infers the concentration of rhamnolipids (μg/ml) in the culture.

Table S2. Ligand correlating phenotypes and corelated receptor.

	Phenotype	Ligand		Inferring Receptor[a]
		Chimeric, 3,5-diMeDβM	Pili only, $SFEG_4OH$	
High cdG phenotypes	Biofilm	Inhibit	No effect	LecA, not pili.
	SCVs	Inhibit	No effect	LecA, not pili.
	Rugose colony EPS	Inhibit	No effect	LecA, not pili.
	Pellicles	Inhibit	No effect	LecA, not pili.
Low cdG phenotypes	Twitching Swarming	Inhibit	Inhibit	Pili only[b]
	Elastase	Decrease	Decrease	Pili only[c]
	Pyocyanin	Decrease	Decrease	Pili only[c,d] Not LecA as low cdG promote pyocyanin.
Both high and low	Rhamnolipids	Increase	Increase	Pili, and may be LecA[e]

[a] Protein receptors (Pili and Lectin A) responsible for controlling phenotypes. [b] low cdG promotes motility[5]. [c] Pili surface sensing induce Vfr, which promotes quorum sensing that results in promotion of elastase and rhamnolipids[6-8]. [d] low cdG promotes pyocyanin[9]. [e] low cdG promotes rhamnolipids[9].

2.Materials and Methods

Bacterial strains and bacteriophage used in paper:

Freezer stocks of all strains (Supplementary for Bacterial strains) were stored at -80 ^{o}C in lysogeny broth (LB) with ~20% glycerol. All strains were grown in LB (10 g/l tryptone, 5 g/l yeast extract, and 10 g/l NaCl) or Mueller-Hinton Broth MHB (beef infusion solids, 2.0 g/l. casein hydrolysate, 17.5 g/l. starch, 1.5 g/l) at 37 ^{o}C with shaking at 250 rpm. All biofilm inhibition, dispersion assays were performed in LB or MHB medium, and plates were incubated at 37 ^{o}C under static conditions.

Table S3. Bacterial strains and bacteriophage

Strain	Number	Source
P. aeruginosa PAO1 wild type		Dr. Guirong Wang (Upstate Medical University)
PAO1-EGFP		Dr. Guirong Wang (Upstate Medical University)
Δ*pilA* knock out mutant		Dr. Rob Lavigne (Katholieke Universiteit Leuven)
pilT transposon mutant	PA0395	PAO1 transposon mutant library (Manoil lab)
LecA transposon mutant	PA2570	PAO1 transposon mutant library (Manoil lab)
P. aeruginosa pili source strain	PA1244N3 (pPAC46)	Dr. Castric (Duquesne University) & Dr. Horzempa (Liberty University)
PAO1k, phage sensitive strain *wt* PAO1		Dr. Rob Lavigne (Katholieke Universiteit Leuven)
Lytic Phage ϕKMV specific for PAO1k		Dr. Rob Lavigne (Katholieke Universiteit Leuven)

General Materials

Stock solutions of 10 mM of 3,5-diMeDβM, 3,5-diMeDβC, SFEG$_4$OH, and 10 mg/ml of tobramycin sulfate were prepared in sterile (autoclaved) water. Stock solutions of agent (3,5-diMeDβM, and 3,5-diMeDβC), and non-autoclavable reagents were further filtered through cellulose acetate syringe filter (0.2 µm pore, Millipore). All overnight cultures of bacteria were grown in Lysogeny Broth, Miller (LB-Miller, 10 g/l tryptone, 5 g/l yeast extract, 10 g/L NaCl). All biofilm assays were performed by immersing the pegs of a modified polystyrene microtiter lid of MBEC™ plates (Innovotech, Alberta, Canada) in M63 medium. All the optical density/absorbance measurements were carried out on Biotek ELx800™ absorbance microplate reader (BioTek Instruments, Inc. Winooski, VT).

Blood agar for revealing small colony variants: To prepare the blood agar gel containing our agents for visualizing small colony variants (SCVs), 1.75 g of premixed powder (Thermo Scientific™ Oxoid™ Columbia Blood Agar Base) containing peptone mixture 25.1 g/l, soluble starch 1.0 g/l, sodium chloride 5.0 g/l, agar 12.0 g/l was mixed and autoclaved with 100 mL of sterile water. The solution was cooled briefly without solidifying and followed by adding 5 mL of sheep blood (Hemostat) and mixed gently by swirling. Portions of 20 mL of this solution was poured into a 50 mL falcon tube, followed by adding 170 µL of a stock solution of agents 3,5-diMeDβM/βC (10 mM) to obtain gel solutions containing 85 µM of 3,5-diMeDβM/βC. The 20 mL gel solutions were poured into petri dishes (10-cm diameter) and solidified for inoculation and visualizing colonies of SCVs.

Crystal violet dye-based biofilm assay[10]

An overnight culture (100 µL) of bacteria in LB was diluted in 10 mL of M63 and incubated to reach an OD_{600} value of ~0.1. The bacterial culture (150 µL) was added to the wells of MBEC™ microtiter plate, followed by predetermined volumes of 3,5-DiMeDβM/βC stock solution for targeted concentrations (6 wells/concentration). The MBEC™ plates were incubated under stationary conditions at 37 ℃ for 24 h. After incubation, the pegs were transferred and immersed into 96 wells containing sterile water (200 µL) twice to briefly rinse the biofilms to remove unattached or loosely attached bacteria and were dried at 37 ℃ for 30 min. The peg-attached biofilms were immersed into 96 wells containing crystal violet (CV) dye solution (150 µL, 0.1%) at ambient temperature for 30 min, to stain the biofilms. The CV-stained pegs were then washed twice with sterile water (200 µL). To solubilize the CR stains, 150 µL of 30% acetic acid solution was added to wells, and the pegs was immersed in the wells, the plates were shake on a microplate mixer (Scilogex MX-M) at 100 rpm for 15 min. The amount of biofilms was inferred and quantified by measuring the OD_{600} of the 150 µL acetic acid solution on plate reader. The absorption from stained pegs containing just M63 medium was subtracted from pegs treated with agents.

Confocal microscopy of biofilms and image acquisition[1, 11]

In a 24-well microtiter plate, biofilms were grown by placing 100 µL of *wt* PAO1 culture with and without tobramycin, and with and without 85µM 3,5-diMeDβM/βC, on polystyrene coupons (roughly 3/8 in. × 3/8 in.) that were cut from a polystyrene petri dish. The Saran-wrapped plate was then incubated at 37 ^{o}C without shaking. Each polystyrene coupon was then washed gently by immerging into saline twice and then placed on a microscope cover glass (50 x 24 mm, No. 2, Fisher Scientific, Pittsburgh, PA). All microscopy images were acquired using a Zeiss LSM confocal laser scanning microscope (Carl Zeiss, Germany) for monitoring Green Fluorescent protein (GFP) and propidium iodide (PI) fluorescence.

Colony morphology assay[12]

Overnight cultures of wt PAO1 (100 µL) was diluted with 10 mL MHB medium containing 0.3 ug/ml tobramycin with and without 85 µM of 3,5-diMeDβM or 3,5-diMeDβC and cultured further for 6 h. These cultures (10 µL) were inoculated on a 1 wt% LBNS agar gels containing 0.3 µg/ml tobramycin with and without 85 µM of 3,5-diMeDβM or 3,5-diMeDβC. The agar plates were incubated at 37°C for 3 days, and images of the colonies were taken. The agar gels were prepared by autoclaving 1 wt% bacto agar in LBNS medium. The gel solution was cooled to about 60 ºC, and poured into Falcon tubes for 20 mL, and

supplemented with filtered solutions of Congo red (40 μg/ml), Coomassie brilliant blue dye (20 μg/ml), and tobramycin (0.3 ug/ml), 3,5-diMeDβM or 3,5-diMeDβC (85 μM). The falcon tubes were closed, gently mixed, and then the agar solution was poured into polystyrene petri dishes (10 cm-diameter, Fisherbrand™), and solidified at room temperature.

Characterization of rugose and smooth colony morphology of PAO1 caused by 0.3 μg/ ml tobramycin with and without 85 μM 3,5-diMeDβM/βC[13-14]

The overnight culture (100 μL) of *wt* PAO1 was diluted in 10 mL LB medium and treated with and without 0.3 μg/ ml tobramycin with and without 85 μM 3,5-diMeDβM/βC 37 ^{o}C with shaking at 250 rpm for 6 h. The 10 μL of these cultures were spotted on 1 % bacto agar plates containing 20 mL colony morphology assay medium (1% tryptone; Bacto), with 40 μg/ml Congo red (CR) and 20 μg/ml Coomassie brilliant blue dyes (EMB). Colonies were incubated at 37°C for 3 days.

Pellicle formation and quantification[14]

Pellicle formation assay was adopted as previously described. Briefly, overnight culture (100 μL) of *wt* PAO1 were inoculated in borosilicate glass tubes (18 mm by 150 mm) containing 10 mL of MHB supplemented with and without 0.3 μg/ml tobramycin, 85 μM 3,5-diMeDβM/βC. Tubes were incubated without shaking at 37°C for 3 days. Pellicle formed at the air-liquid interface was resuspended in 1.5 mL PBS buffer. Congo red stock solution (10 mg/ml) was added to a final concentration of 40 μg/ml and incubated with shaking for 2 h at 37°C. Following this incubation, the bacteria were pelleted via centrifugation (6000 rpm, 15 min); and the absorbance (490 nm) of 200 uL of the supernatant was measured on plate reader. The PBS with 40 μg/ml Congo red was used as the reference to determine the amount of Congo red bound to pellicles.

Quantification of biofilm bacteria and mass[1, 11]

We quantified the killing of bacteria in biofilms under different chemical treatment (tobramycin and 3,5-diMeDβM/βC) by CFU counting, and measured biofilm mass by CV dye assay. An overnight culture (100 μL) of bacteria in LB was diluted in 10 mL of M63 and subcultured to reach an OD_{600} value of ~0.1. The bacterial culture (150 μL) was added to the wells of MBEC™ microtiter plate, followed by predetermined volumes of 3,5-diMeDβM/βC stock solution for targeted concentrations (6 wells/concentration). The 24 h-old biofilm on pegs were transferred to wells of saline briefly, and then transferred to wells containing 150 μL of fresh M63 medium containing of tobramycin, 3,5-diMeDβM or 3,5-diMeDβC with targeted concentrations and were incubated for an additional 0-24 h at 37 ^{o}C. The pegs were washed with saline and clipped with sterile plier. Each condition consisting of 6 pegs was sonicated in 5 ml of saline and serially diluted in saline followed by plating on LB agar plates for 24 hours for counting colony forming units. The amount of the biofilms on pegs were measured by using the crystal violet staining method described in biofilm inhibition assay. The bacterial count (CFU/ml) was normalized by amount of biofilm.

Hemolysis assay[15]

Single donor Human Red Blood Cells (Innovotech) suspension (300 μL) was mixed with different volumes of 3,5-diMeDβM/C to reach final concentrations of 0-500 μM in PBS buffer, respectively. mix 300 μL of RBC suspensions with of 30 μL of Triton X (1%) and PBS, respectively as Positive and negative controls After incubation at 37℃ with 100 rpm shaking for 1 hthe samples were then centrifuged for 2 min at 2000 rpm. The absorbance of 200 μL supernatant was recorded at 540 nm. % Hemolysis was calculated as $(OD_{sample} - OD_{negative\ control})/(OD_{positive\ control} - OD_{negative\ control}) \times 100\%$. Experiments were performed in triplicate.

Critical Aggregation Concentration (CAC) assay[16]

A 3,5-diMeDβM/βC stock solution (10 mM) was diluted in PBS buffer at various concentrations (0-200 μM). An ethanol solution (1 μL) of Nile red (2.5 mM) was added to 1 mL assay solution, and the fluorescence emission was measured on a using Synergy 2 multimode microplate reader an excitation

wavelength of 550 nm after 20 minutes of incubation at 37℃. Fluorescence intensity was recorded at 635 nm. Experiments were performed in triplicate.

Bacterial Growth as 3,5-diMeDβM as the sole carbon source[17]

Wild type *P. aeruginosa* strain was grown on 1.5 % agar gel containing modified M9 minimal salts, 3.5 g of $NaNH_4HPO_4 \cdot 4 H_2O$, 7.5 g of $K_2HPO_4 \cdot 3H_2O$, 3.7 g/liter KH_2PO_4, and 0.1% (vol/vol) trace salts solution. One liter of trace salts solution contained 2.86 g of $FeCl_3 \cdot 6H_2O$, 1.98 g of $MnCl_2 \cdot 4H_2O$, 3.31 g of $CoCl_2 \cdot 6H_2O$, 1.47 g of $CaCl_2 \cdot 2H_2O$, 0.17 g of $CuCl_2 \cdot 2H_2O$, and 0.61 g of $ZnCl_2$. Combinations of either 85μM of 3,5-diMeDβM or 2% glucose were provided as the sole carbon source. Growth of bacterial colonies was monitored at 37°C for 1-3 days.

Twitching assays[18-19]

Subsurface twitching assays were modified from previously reported procedures. Briefly, 100 μL of overnight culture of PAO1-EGFP was diluted in 900 μL of Luria Bertani broth without NaCl (LBNS). Sterile pipette tips (10 μL) were dipped in this LBNS suspension and were then stabbed through a one-day-old 1% LBNS agar, with and without 85 μM of 3,5-diMeDβM or 3,5-diMeDβC, to inoculate bacteria at bottom of the agar gel. Plates were incubated upright (not inverted) in an incubator at 37 ℃ for 5 days, and images of the plates were taken under ultraviolet light (Accuris™ E3000 UV Transilluminator - 302nm) in dark room.

Gel electrophoresis of sheared surface proteins from wild type *P.* aeruginosa[20]

The levels of pili on bacterial surface were analyzed by gel electrophoresis as described previously with slight modifications[20]. The *wt* PAO1 and *transposon mutant pilT::Tn* were streaked on LB agar plates with and without 85μM 3,5-diMeDβM/βC and incubated at 37 °C for ~ 16 h. The bacterial cells were scraped from the agar gels with glass coverslips and resuspended in 5 mL of phosphate buffered saline, pH 7.0, with and without 85μM 3,5-diMeDβM/βC, respectively. The solutions were centrifuged at 6000 rpm for 15 min to collect bacterial cells. The collected bacterial pellet was resuspended in 1 ml PBS buffer, and vortexed for 30 s to shear surface proteins from suspended bacterial cells. The suspensions were transferred to three separate 1.5 ml Eppendorf tubes and centrifuged at 11,688 x *g* for 5 min to pellet the bacterial cells. The supernatant was transferred to fresh tubes and centrifuged at 11,688 x *g* for 20 min to further pellet remaining cells. The supernatants were collected in new tubes, mixed with 1:10 volume of 5 M NaCl and 30% (w/v) polyethylene glycol (PEG 8000; Sigma-Aldrich), and incubated on ice for 90 min, to precipitate the surface proteins. The solutions were centrifuged at 11,688 x *g* for 20 min, to collect the aggregated proteins. The protein pellets were resuspended in 100 μL PBS buffer and the amount of total surface sheared protein was determined by Bradford assay that resulted for 120 μg/ml protein mass for *wt* PAO1 without 3,5-diMeDβM/βC treatment, and 136 μg/ml for *wt* PAO1 with 3,5-diMeDβM/C treatment, which were diluted to 120 μg/ml; and 470 μg/ml for *transposon mutant pilT::Tn* without 3,5-diMeDβM/βC treatment, which was diluted to 321 μg/ml, and 321 μg/ml for *transposon mutant pilT* with 3,5-diMeDβM/βC treatment. The protein samples (80 μL) were mixed with 150 μL of 1X sodium dodecyl sulfate (SDS) sample buffer (125 mM Tris, pH 6.8, 2% β-mercaptoethanol, 20% glycerol, 4% SDS and 0.001% bromophenol blue), and boiled for 10 min, cooled to room temperature; 20 μL of the sample were loaded to for separation on 15% SDS–polyacrylamide gel electrophoresis (SDS–PAGE) gels. Proteins were visualized by staining with Coomassie brilliant blue.

Quantification of phage titer, plaque forming unit (PFU), by top overlay method[4]

All phage counts were quantified by phage titer using top overlay method to report the plaque forming units.[4] *Top agar preparation.* Mix 3 mL of warm top agar solution (7 g/l agar, 0.5 g/l sodium chloride, 10 g/l tryptone, 5 g/l yeast extract in sterile water) and 200 μL of LB culture of PAO1k (OD 0.6); spread this mixture solution on 1.5% LB agar plate (LB-Miller, 10 g/l tryptone, 5 g/l yeast extract, 10 g/L NaCl ,15 g/l agar, 10-cm in diameter). Cool for 15 min in biosafety level 2 hood to solidify the gel. Serial dilutions of supernatant of containing phage were prepared in LB-Mg^{2+}. Nine or six drops of 10 μL of the diluted

supernatant were placed on the top agar plates to observe plaques formation on bacterial lawn after 12 h. The number of plaques were counted and reported as plaque forming units per ml of sample.

Lysis of bacterial cells by bacteriophage ϕKMV[4, 21-22]

The overnight culture (100 μL) of (PAO1k, Δ*pilA*, *pilT::Tn*) was diluted in 10 mL LB medium with $MgSO_4$ (10 mM) and was grown to mid-log phase (OD=0.5) with and without 85 μM 3,5-diMeDβM or 3,5-diMeDβC at 37℃ at 250 rpm. These cultures (OD_{600} = 0.5) were infected with φKMV phage (PFU/ml = 3.4 × 10^9). The optical density (OD_{600}) of bacterial culture was monitored over 12 h. The clear bacterial lysate (OD < 0.1) is considered as the end point for lysis experiment.

Direct binding of fluorescent ligands to LecA[23]

The fluorescent ligand (βGal-aryl-Dansyl) were dissolved in water to a final concentration of 3 mg/ml. 2 mg LecA (Sigma) was dissolved in 1 mL of 0.1M Tris-HCl pH 7.5 supplemented with $CaCl_2$ (6 μM). The solution of fluorescent ligand (βGal-aryl-Dansyl, 200 nM) is mixed with different concentrations of LecA solution (0-100 μM). After incubation for 1 h at room temperature in dark, fluorescence polarization was measured by using Edinburgh FLS9801 Spectrometer, with the samples being illuminated with vertically polarized light at 330 nm (for βGal-aryl-Dansyl). The vertical and horizontal fluorescence components of emission 570 nm were recorded, and the fluorescence polarization was calculated and data were analyzed using a four-parameter fit model.

Competitive binding of ligands to LecA[23]

To a solution of LecA (final concentration: 20 μM) and fluorescent ligand (final concentrations of βGal-aryl-Dansyl: 200 nM) in 0.1M Tris-HCl pH 7.5 supplemented with $CaCl_2$ (6 μM), serial dilutions (0.1 μM to 140 μM) of test compounds (SFβM/C, rhamnolipids, $SFEG_4OH$ and 3,5-diMeDβM/βC) were done. The mixture samples were incubated for 6 hours at room temperature. The fluorescence polarization was measured using Edinburgh FLS9801 Spectrometer. During measurement, the samples were illuminated with vertically polarized light at 330 nm (for βGal-aryl-Dansyl) and vertical and horizontal fluorescence components were measured, and the fluorescence polarization was calculated and data were fitted with four parameter variable slope model.

Elastase assay[18, 24]

Elastase activity in *P. aeruginosa* culture was determined by an elastin Congo red (ECR) assay with modifications[18, 24]. Briefly, 100 μL of bacteria from overnight cultures in MHB were diluted in fresh MHB to an OD_{600} of 0.01 with and without 3,5-diMeDβM or 3,5-diMeDβC, $SFEG_4OH$ (final concentration: 85 μM). After culturing for 18 h at 37°C with shaking, culture supernatants were filtered (0.45-μm pore-size filter). Triplicates of culture filtrates (100 μL) were added to 1 mL of Tris buffer (0.1 M Tris, pH 7.2, 1 mM $CaCl_2$) containing 20 mg of Elastin-Congo red (Sigma Aldrich). The glass tubes were incubated for 6 h at 37°C with shaking at 100 rpm and were added with 0.1 ml of 0.12 M EDTA and placed on ice. Insoluble ECR was removed by centrifugation at 16,000 rpm and the OD_{495} of the supernatant was measured.
The elastase activity was reported as OD_{495} (with agent) - OD_{495} (without agent).

Rhamnolipid assay[25]

Quantification of rhamnolipids in planktonic cultures of *P. aeruginosa* was determined by methylene blue-rhamnolipid complex assays by an established assay.[25] In this assay, the positively charged methylene blue form noncovalent complex preferentially with negatively charged surfactants, and does not bind to nonionic surfactants. The methylene blue bound with negatively charged surfactants has an increased partition into the chloroform phase bought in contact with the aqueous solution. The methylene blue in the chloroform phase were measured for its UV absorbance, which infers the concentration of negatively surfactants. Briefly, 100 μL bacteria from overnight cultures in MHB were diluted in fresh MHB to an OD_{600} of 0.01 with and without 3,5-diMeDβM or 3,5-diMeDβC, $SFEG_4OH$ (final concentration: 85 μM). After 18 h at 37°C with shaking, culture supernatants were filtered with 0.45-μm pore-size filter (SCBT, USA). The

filtrate pH was first adjusted to 2.3 ± 0.2 using 1 N HCl. The acidified sample was then extracted with five-fold volume of chloroform. The chloroform extract (4 mL) was transferred to put in contact with 5 mL of a freshly prepared methylene blue aqueous solution (40 µg/ml). The pH of this aqueous solution of methylene blue is pre-adjusted to 8.6 ± 0.2 by adding the 50 mM borax buffer. After 4 mins vortex shaking, the samples were left to stand in dark for 15 min. The bottom chloroform phase (1 mL) was transferred into a cuvette and the absorbance was measured at 638 nm with a UV/Vis spectrophotometer with a reference of blank chloroform. The absorbance values were converted to rhamnolipid concentrations using a calibration curve.

Pyocyanin assay[26]

An overnight culture of PAO1 (100 µL) was added to 10 mL fresh LB medium, 5 mL of which were grown with and without 85 µM 3,5-diMeDβM, or 3,5-diMeDβC, $SFEG_4OH$ for 12 h. The absorbance of bacterial culture was measured at 600 nm (A_{600}), and then centrifuged to remove bacterial pellet. Supernatant (5 mL) was extracted with 3 mL chloroform. The pyocyanin-containing chloroform layer was acidified with 2 mL of 0.1 N HCl to give a pink solution, the absorbance at 520 nm of 200 µL of the solution was measured. The absorbance reading at 520 nm was converted into µg/ml of supernatant with standard curve reported in literature. Pyocyanin levels were expressed as µg/ml of supernatant/A_{600} of culture where A_{600} is absorbance of bacterial culture.

3. Synthesis and Spectral Data

Materials

3,5-dimethylcyclohexanone (mixer of isomers) **1** and β-D-Maltose octaacetate **9**, α-D-cellobiose octaacetate **8** were purchased from Sigma Aldrich. Standard solvents and reagents were purchased from commercial sources (Sigma-Aldrich, Fisher, Acros) and used as received. Solvents were removed *in vacuo* using Bűchi rotary evaporator below 40^{0}C. EMD silica gel 60 F254 pre-coated plates (0.25-mm thickness) were used for TLC. Unless otherwise stated, TLC visualization was done using ceric ammonium molybdate (CAM) stain. ^{1}H-NMR spectra were recorded in deuterated NMR solvents at 400 MHz (^{1}HNMR) and 100 MHz (^{13}CNMR) Bruker instruments, respectively. ^{1}H chemical shifts are reported in ppm relative to $CDCl_3$ δ ppm 7.26, CD_3OD δ ppm 3.31. ^{13}C chemical shifts are reported relative to $CDCl_3$ δ ppm 77.23 and CD_3OD δ ppm 49.0.

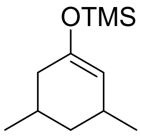

2

To a solution of **1** (2.78 g, 22 mmol) in dry acetonitrile (6 mL) triethylamine (11.5 mL), trimethylchlorosilane (4.1 mL) and sodium iodide (4.63 g, 30.9 mmol) dissolved in acetonitrile (29 mL) were added successively. The reaction mixture was stirred at room temperature. After 4 h, cold water (37 mL) was added and the aqueous solution extracted with cold hexane. The organic layers were combined and evaporated in vacuo to give **2** as a brown oil. Crude product was assessed by NMR and deemed pure enough use crude in next step without further purification (81% yield).

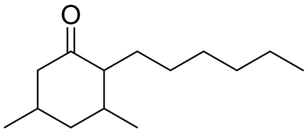

3

A solution of silver trifluoroacetate (2.09 g, 9.45 mmol) suspended in dichloromethane (9 mL) was cooled to -78 ^{o}C and Compound **2** (1.79 g, 9 mmol) was added dropwise. 1-iodohexane (2.0 g, 9.45 mmol) was then added and the reaction was allowed to warm to room temperature then after ten minutes the reaction mixture was filtered and concentrated en vacuo. The crude product was flash chromatographed on silica gel (hexane/AcOEt 95 : 5) to yield **3** as a colorless oil (19% yield). ^{1}H-NMR (400MHz, $CDCl_3$) δ ppm: 0.87 (3H, t), 0.92-0.97 (6H, d), 1.16-1.48 (10H, m), 1.56-1.75 (2H,t), 1.61-1.90 (2H, t), 2.18-2.35 (3H, m)

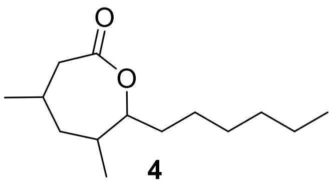

4

A solution of **3** (160 mg, 0.76 mmol) in dry dichloromethane (2 mL) was cooled to 0ºC and m-chloroperbenzoic acid (350 mg, 2.03 mmol) was added. The suspension and trifluoroacetic acid (0.288 mL, 3.76 mmol) was added dropwise. During this operation, the reaction flask was protected from light. Subsequently, the solution was allowed to warm to room temperature. After 16 h, the mixture was diluted with dichloromethane (2 mL) and the organic layer was washed successively with a 10% aqueous solution

of Na_2SO_3 (2 mL), a saturated aqueous solution of potassium carbonate (1 mL) and water (2 mL). Finally, the organic layer was evaporated in vacuo and the product purified by column chromatography (9:1 hexanes: ethyl acetate) to give lactone **4** (46% yield). ^{1}H-NMR (400MHz, $CDCl_3$) δ ppm: δ 0.87 (3H, t), 0.95 (3H, d), 1.00 (3H, d), 1.17-1.59 (12H, m), 1.77-1.92 (2H, d), 2.41-2.54 (2H, dd), 4.29 (1H, td).

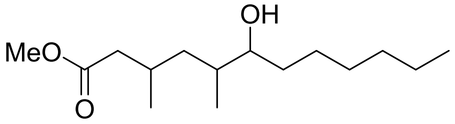

5

Under nitrogen atmosphere, a solution of **4** (1.608 g, 7.58 mmol) in a 10 mM solution in anhydrous methanol (7.1 mL) was stirred overnight at room temperature. Then, the reaction was neutralized using H^+ amberlite resins. After filtration, the filtrate was evaporated in vacuo to give the methyl ester **5** as a clear, oily liquid (87% yield). ^{1}H-NMR (400MHz, $CDCl_3$) δ ppm: 0.84 (3H, t), 0.87-0.92 (6H, d), 0.96-1.24 (13H, m), 2.14-2.19 (2H, dd), 3.45-3.55 (2H, m), 3.65 (3H, s)

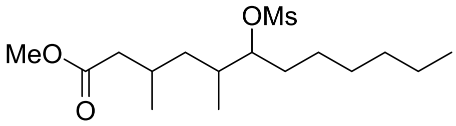

6

A solution of **5** (1.098 g, 0.25 mmol) in dichloromethane (1.5 mL) was stirred on ice. Triethylamine (0.23 mL, 1.6 mmol) and mesyl chloride (655 mL, 0.51 mmol) were added. The mixture was stirred at 0ºC. After 4 h, aqueous $NaHCO_3$ (1N, 1.2 mL) was added and the resulting solution extracted with dichloromethane. The organic layers were combined and evaporated in vacuo and the product purified by column chromatography (9:1 hexanes: ethyl acetate)to give mesylate **6** as a yellowish oil (91% yield). ^{1}H-NMR (400MHz, $CDCl_3$) δ ppm: δ 0.79-0.91 (9H, m), 1.17-1.34 (13H, m), 2.17-2.39 (2H, d), 2.43 (1H, q) 3.42 (3H, s), 4.27 (1H, td).

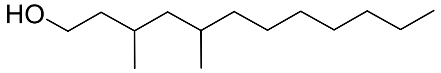

7

To a suspension of LAH (63 mg, 1.7 mmol) in anhydrous diethyl ether (1 mL) at 0ºC, a solution of **6** (68 mg, 0.20 mmol) in anhydrous ether (1 mL) was added. The mixture was brought to room temperature then refluxed for 16 h. Then, the solution was cooled to 0ºC before quenching with water. The resulting aqueous layer was extracted with ether. The combined organic fractions were evaporated in vacuo and the oily residue was flash-chromatographed (hexane/AcOEt 9:1) to yield alcohol **7** as an oil (53% yield). ^{1}H-NMR (400MHz, $CDCl_3$) δ ppm: 0.54-0.74 (9H, m), 0.86-1.54 (18H, m), 3.32-3.40 (2H,t).

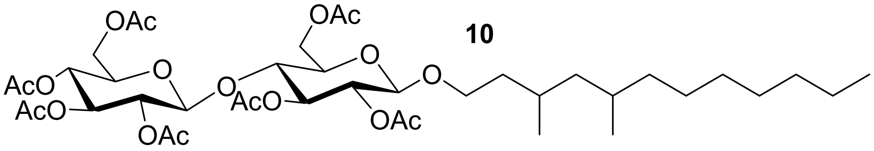

10

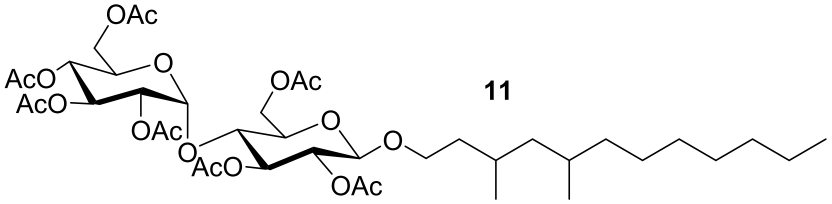

11

The cellobiose octaacetate **8** or maltose octaacetate **9** was dissolved in dichloromethane (10mL) and **7** (2 equivalents) were added. Under vigorous stirring, BF_3. etherate (2 equivalents as compared to maltose or cellobiose monohydrate) was added to this mixture and the reaction mixture was allowed to stir at rt for 12 h. To the reaction mixture was added aq KBr (10%, 25mL) and PhMe (60mL) under stirring. The organic phase was washed twice with aq. KBr (10%, 2×25mL), once with aq. $NaHCO_3$ (5%, 25mL) and twice with H_2O (2×25mL). Using a gradient elution (100 % hexane to 35 % ethyl acetate in hexane) the crude product was then purified through a silica column to yield white powder.

10 (0.20 mg, 30% yield). ^{1}H (400 MHz, CDCl3) δ 5.16-4.98 (m, 3H), 4.85 (q, *J1-2* = 8.4 Hz, 2H), 4.48-4.29 (m, 4H), 4.06 - 3.97 (m, 2H), 3.82 - 3.69 (m, 2H), 3.66 -3.50 (m, 2H), 3.44 - 3.34 (m, 1H), 2.07-1.93 (s, 7 X 3H), 1.56 -1.32 (m, 2H), 1.20 (br, s, 18 H), 0.83 (m, 9H).

11 (0.18 mg, 12% yield). ^{1}H (400 MHz, CDCl3) 5.43-5.36 (m, 2H), 5.25 (t, 1H), 5.07 (t, 1H), 4.97-4.79 (m, 2H), 4.53-4.42 (m, 2H), 4.29-4.20 (m, 2H), 4.07-3.85 (m, 4H), 3.70-3.65 (m, 1H), 3.53-3.44 (m, 1H), 2.14-2.01 (s, 7 x 3H), 1.66-1.16 (m, 18 H), 0.89 (br, s, 9H).

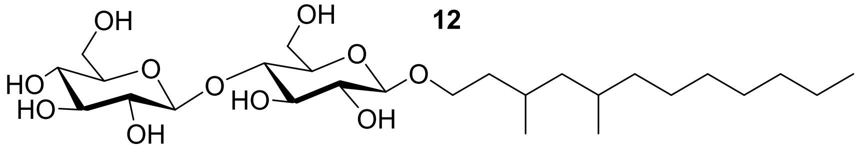

12

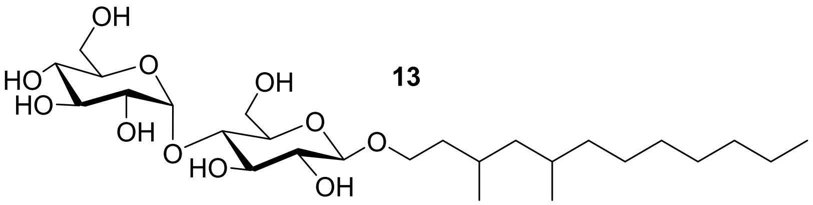

13

Zemplen deacetylation was used to deprotect the acetylated glycosides. Briefly, acetylated glycosides were treated with methanolic solution of MeONa (10 mM) followed by neutralization (pH ~ 7) over H+ amberlite resins. The product was filtered and dried under vacuum overnight to get white powder.

12, (9.3 mg, 75%) ^{1}H NMR (300 MHz, MeOD) δ 4.38 (d, *J* = 7.8 Hz, 1), 4.24 (d, *J* = 7.8 Hz, 1H), 3.83 (br, s, 4H), 3.62 (dd, *J1-3* = 12.0 Hz, *J1-2* = 5.1 Hz, 1H), 3.56-3.44 (m, 3H), 3.37-3.27 (m, 5H, overlapping with MeOD), 1.25 (br, s, 18 H), 0.86 (m, 9H).). ^{13}C NMR (75 MHz, CD3OD): δ 102.26, 103.23, 76.72, 76.42, 75.11, 75.04, 73.54, 73.45, 69.97, 67.86, 61.08, 60.54, 37.68, 36.56, 31.67, 29.83, 29.77, 29.65, 29.09, 26.75, 26.65, 22.32, 19.23, 18.48, 13.2.

HRMS (ESI) m/z: Calcd. (C26H50 O11)Na+: 538.68; Found: 537.327526.

13, (6mg, 97%) ^{1}H NMR (300 MHz, MeOD) δ 5.2 (br, s, 1H), 4.27 (d, 1H), 3.95-3.92 (m, 4H), 3.88-3.43 (m, 6H, overlap with CD3OD peak), 3.27-3.20 (m, 4H), 1.79-1.13 (m, 18H), 0.90- 0.87 (m, 9H). ^{13}C NMR (75 MHz, CD3OD): δ 101.54, 101.52, 79.98, 76.50, 75.21, 73.69, 73.35, 72.78, 70.10, 61.36, 60.81, 36.57, 31.67, 29.83, 29.77, 29.65, 29.09, 26.75, 26.65, 22.32, 19.23, 18.48, 13.2.
HRMS (ESI) m/z: Calcd. (C26H50 O11)Na+: 538.58; Found: 537.327637.

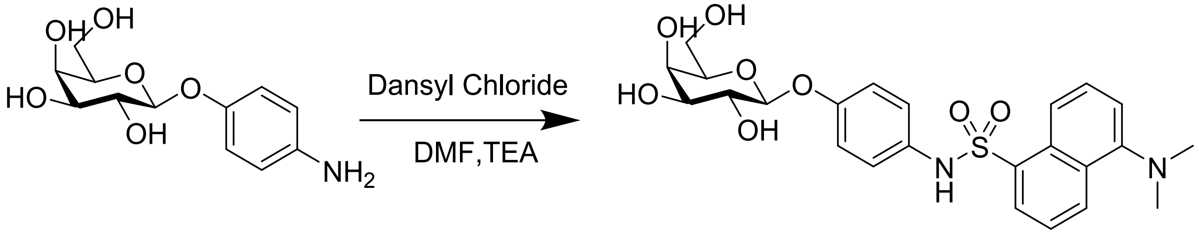

Figure S16: Synthesis scheme for a fluroscent probe, βGal-aryl-Dansyl
4-Aminophenyl β-D-galactopyranoside (0.12 g, 1.2 mmol) in anhydrous DMF (2 mL) was added to Et_3N (0.53 g, 0.53 mmol) in anhydrous DMF (5mL) at 0 ºC. To this solution was added dansyl chloride (0.41 g, 1.5 mmol). After stirring at 0 ºC for 2 hours, the mixture was concentrated, and the residue obtained was subjected to the purification by column chromatography DCM:MeOH 9:1 to yield a yellow solid product (0.44 g, 72 %). ^{1}H NMR (400MHz, $CDCl_3$): δ 8.47 (d, 1 H, dansyl), 8.24 (d, 1 H, dansyl), 8.20 (d, 1 H, dansyl), 7.65-7.69 (m, 2 H, dansyl), 7.39 (d, 1 H, dansyl), , 6.92–6.81 (m, 4H, ArH), 4.75 (d, J =7.7 Hz, 1H), 4.37 (s, 2H, CH_2NHR), 3.69 (d, J = 3.1 Hz, 1H), 3.58–3.46 (m, 5H), 2.84 (s, 6H,-CH_3).

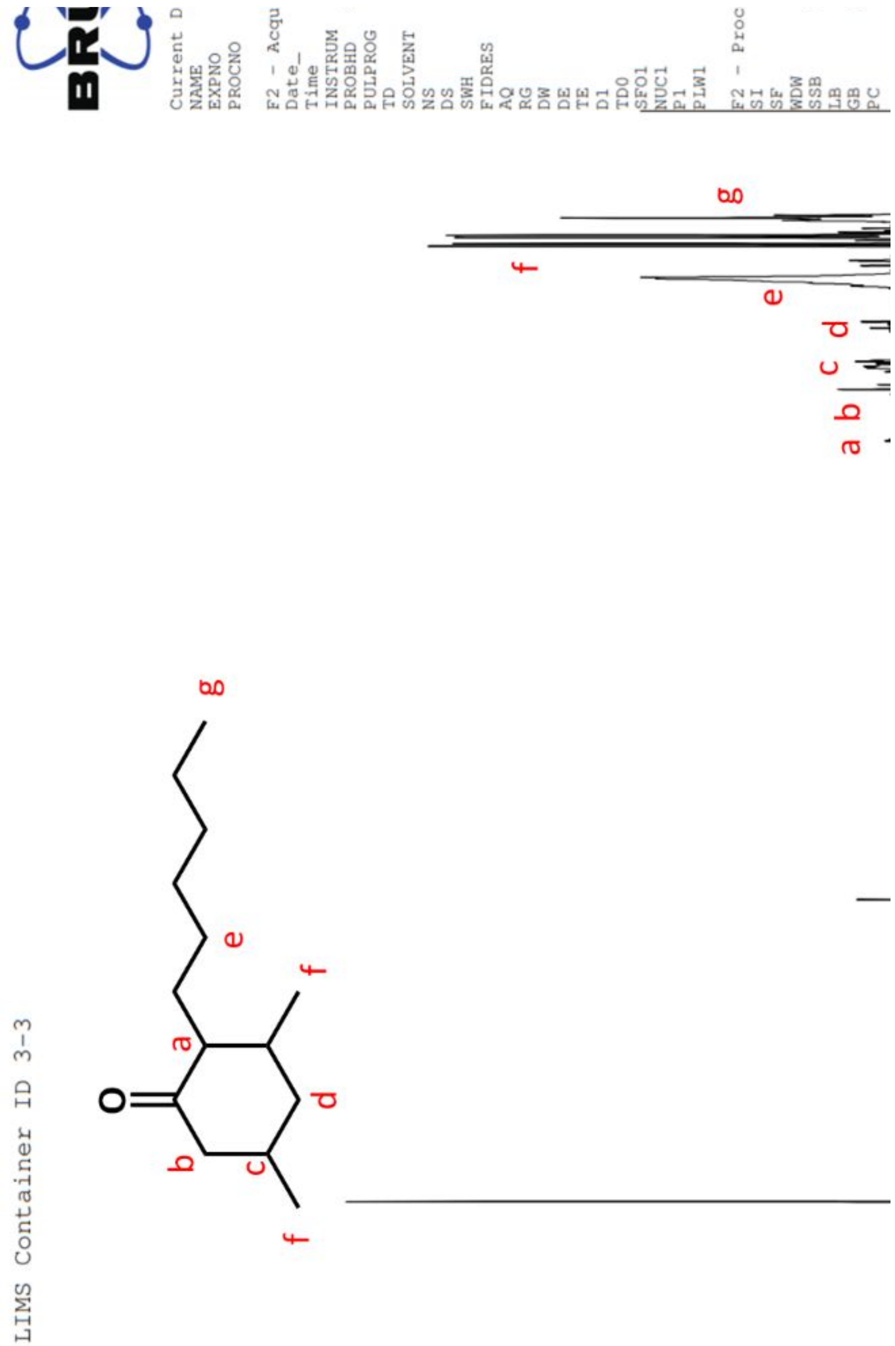

Figure S17: ^{1}H NMR spectrum of **3** in $CDCl_3$

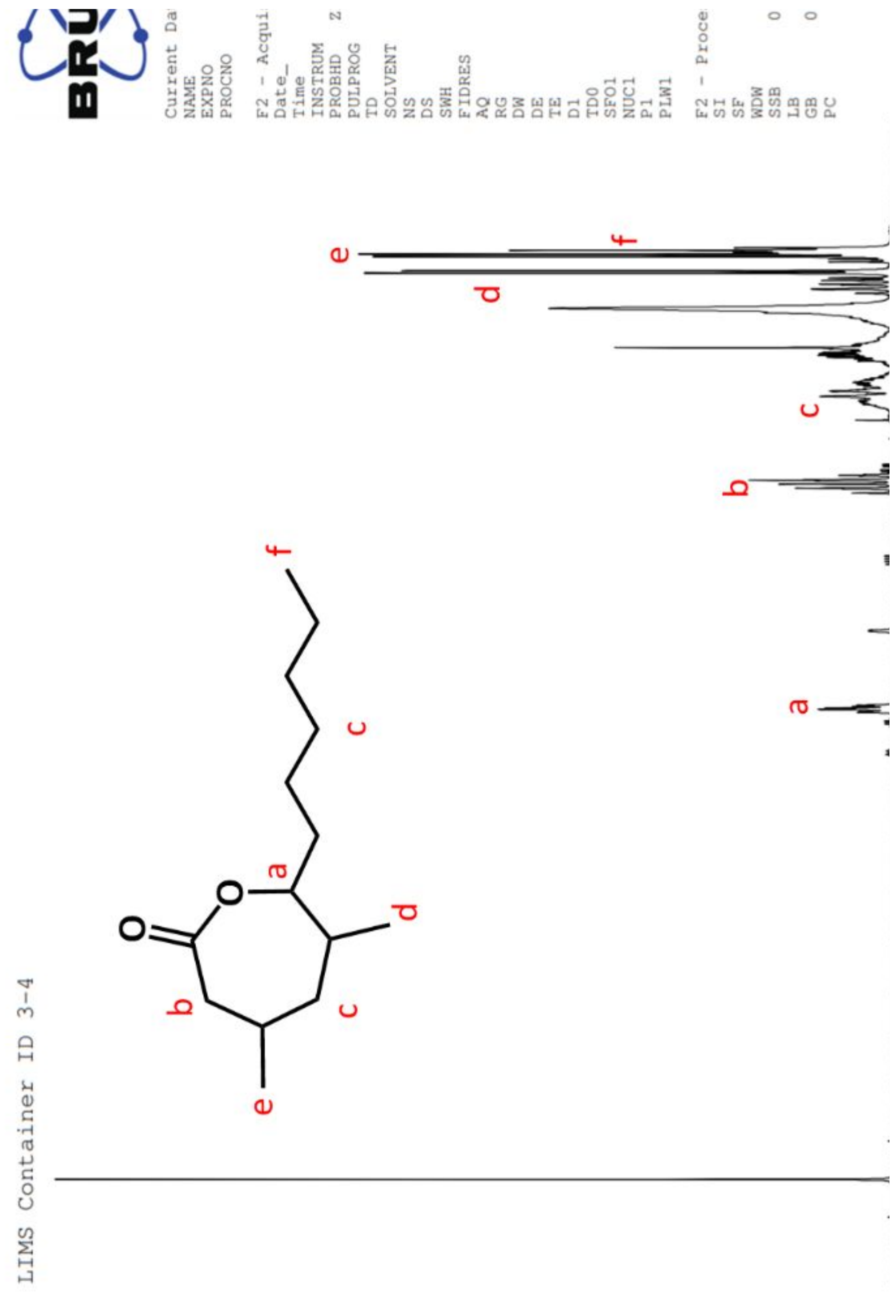

Figure S18: ^{1}H NMR spectrum of **4** in $CDCl_3$

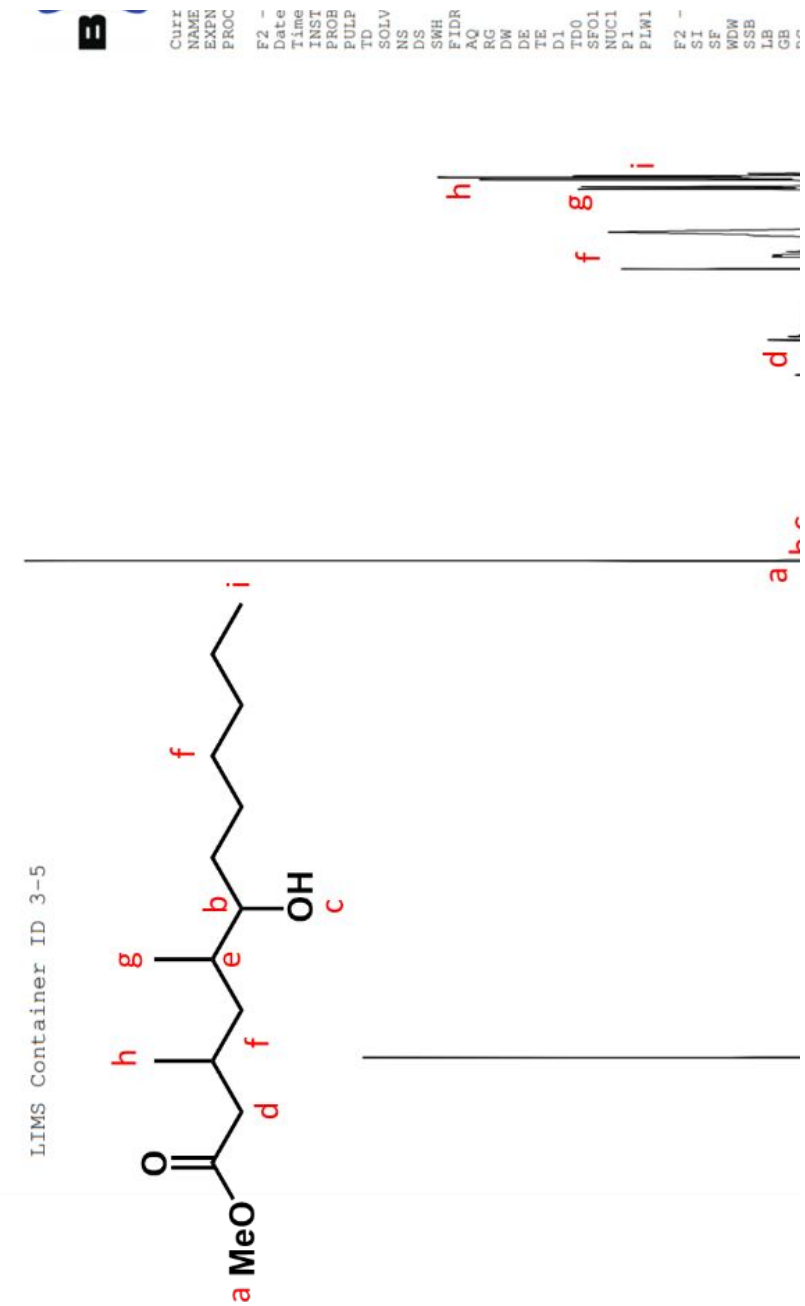

Figure S19: ^{1}H NMR spectrum of **5** in $CDCl_3$

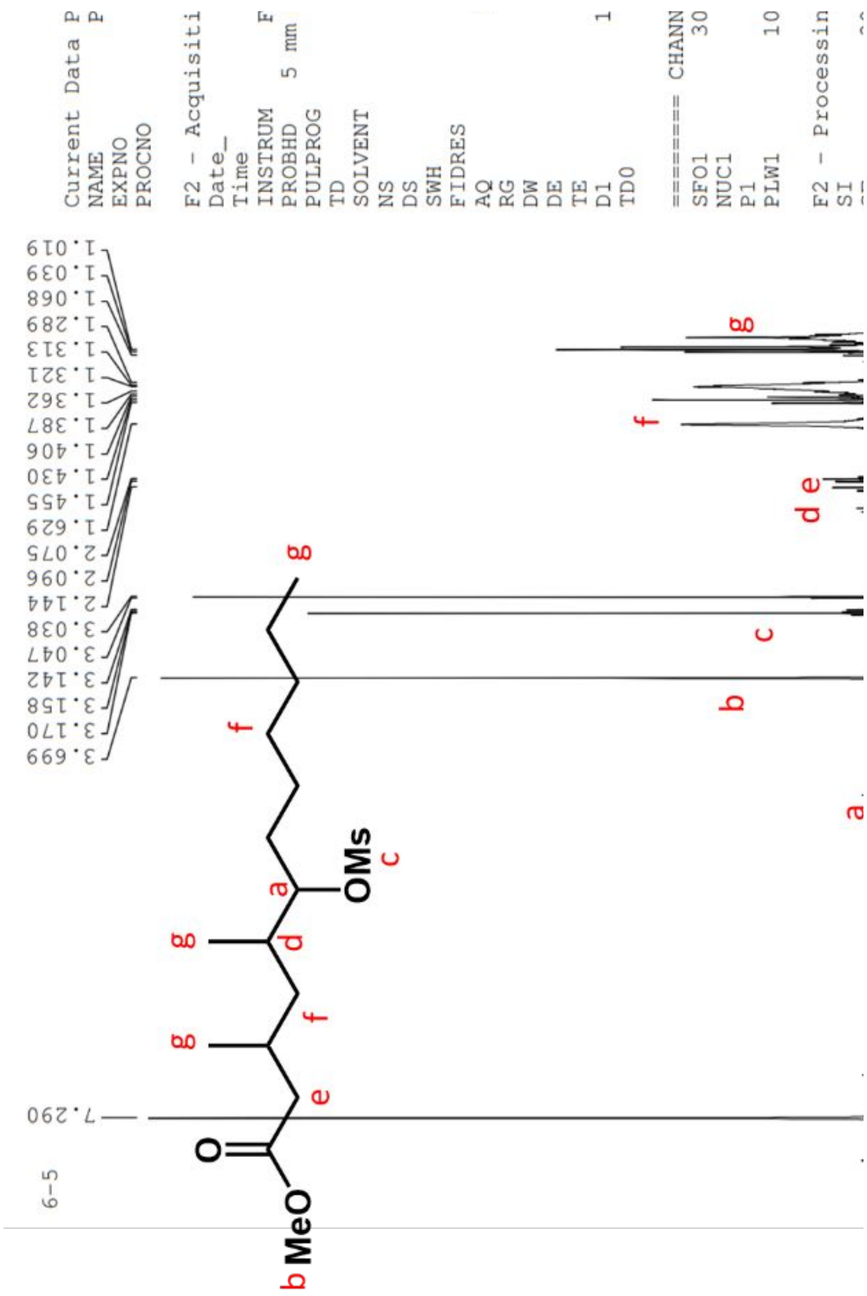

Figure S20: ^{1}H NMR spectrum of **6** in $CDCl_3$

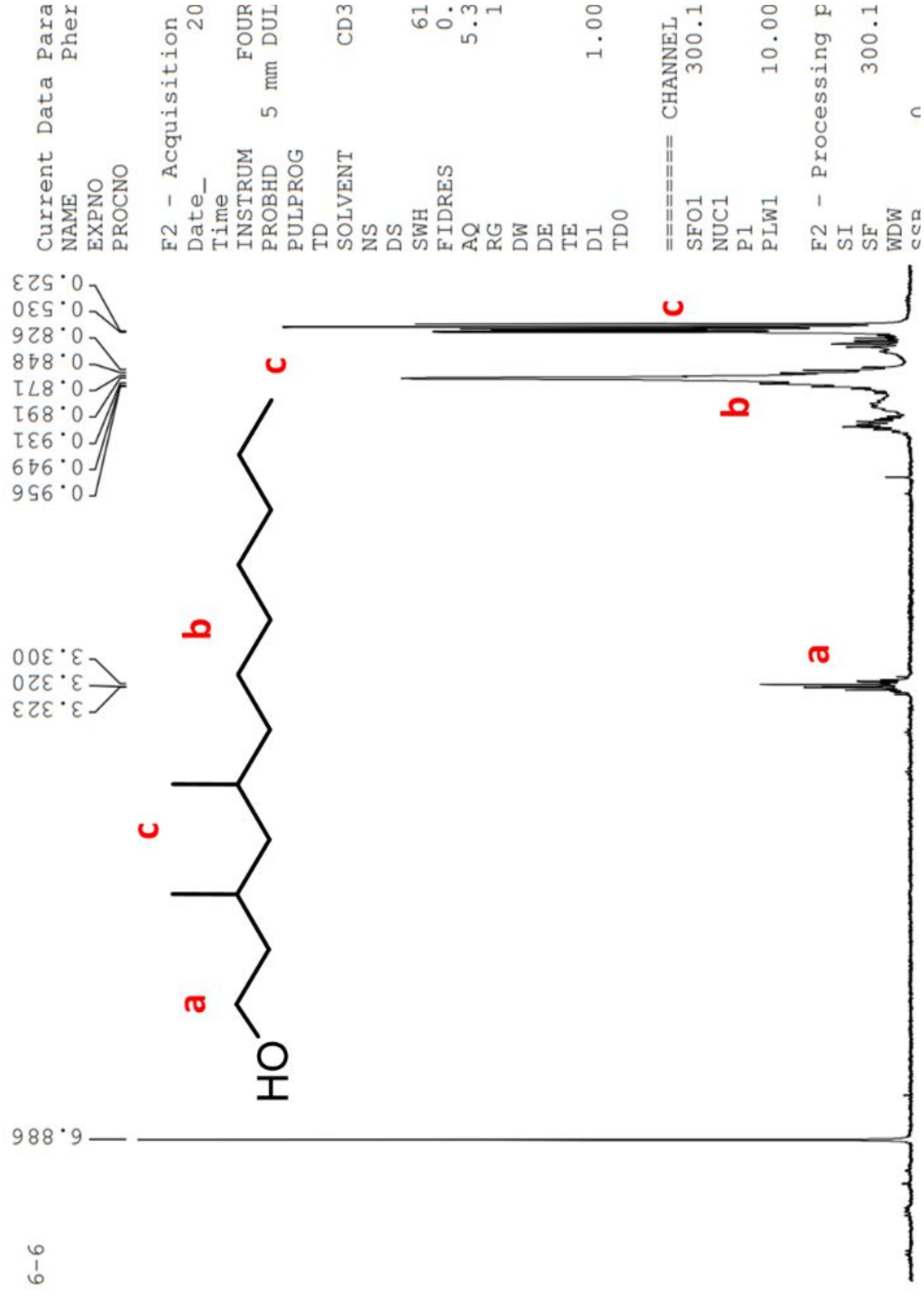

Figure S21: ^{1}H NMR spectrum of **7** in $CDCl_3$

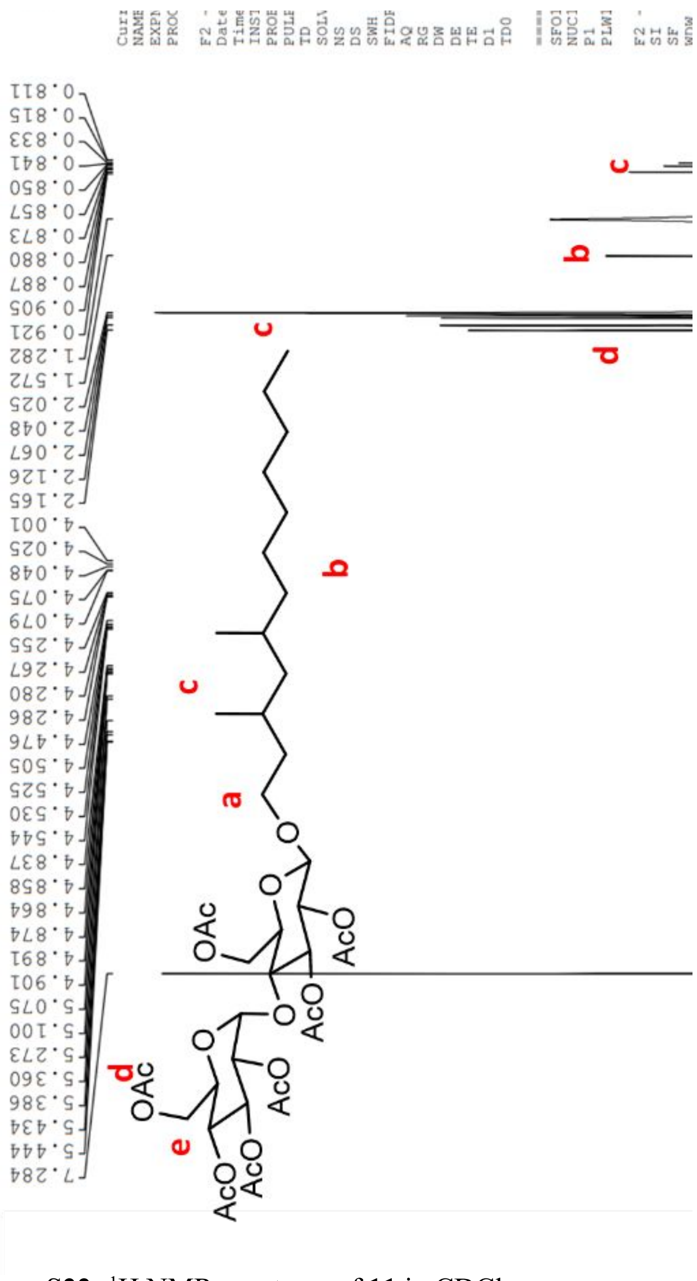

Figure S22: ^{1}H NMR spectrum of **11** in $CDCl_3$

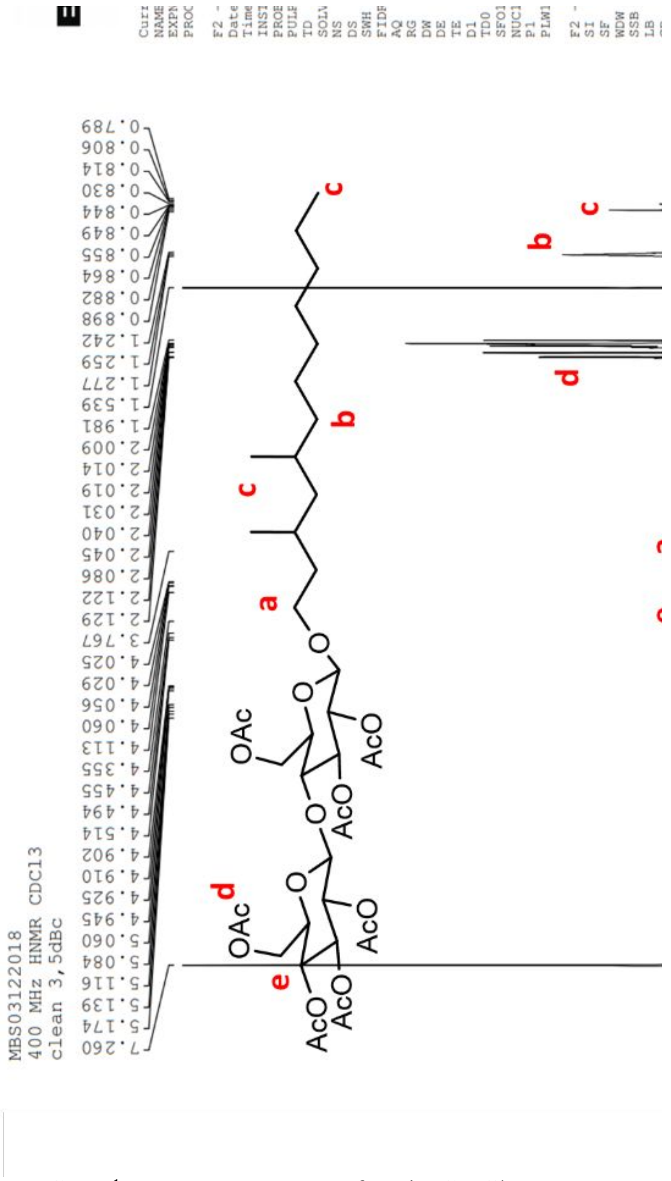

Figure S23: ^{1}H NMR spectrum of **10** in $CDCl_3$

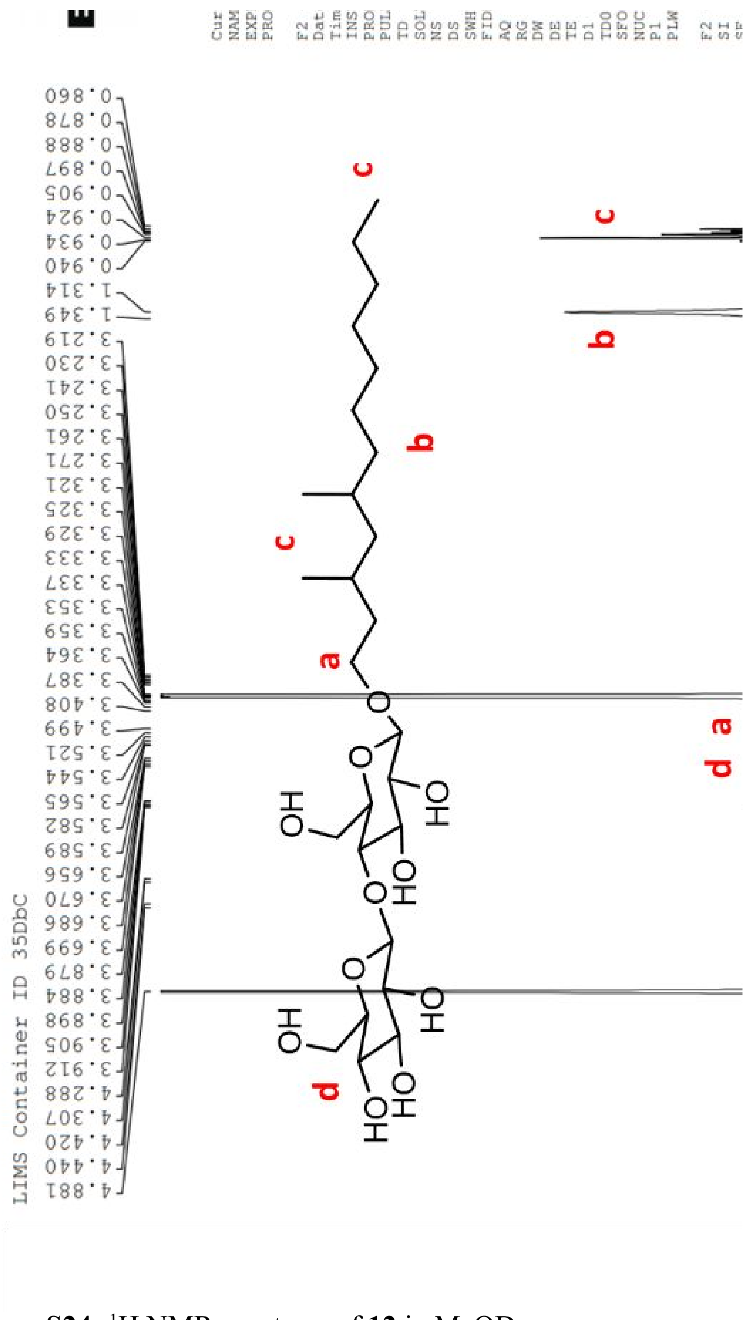

Figure S24: ^{1}H NMR spectrum of **12** in MeOD

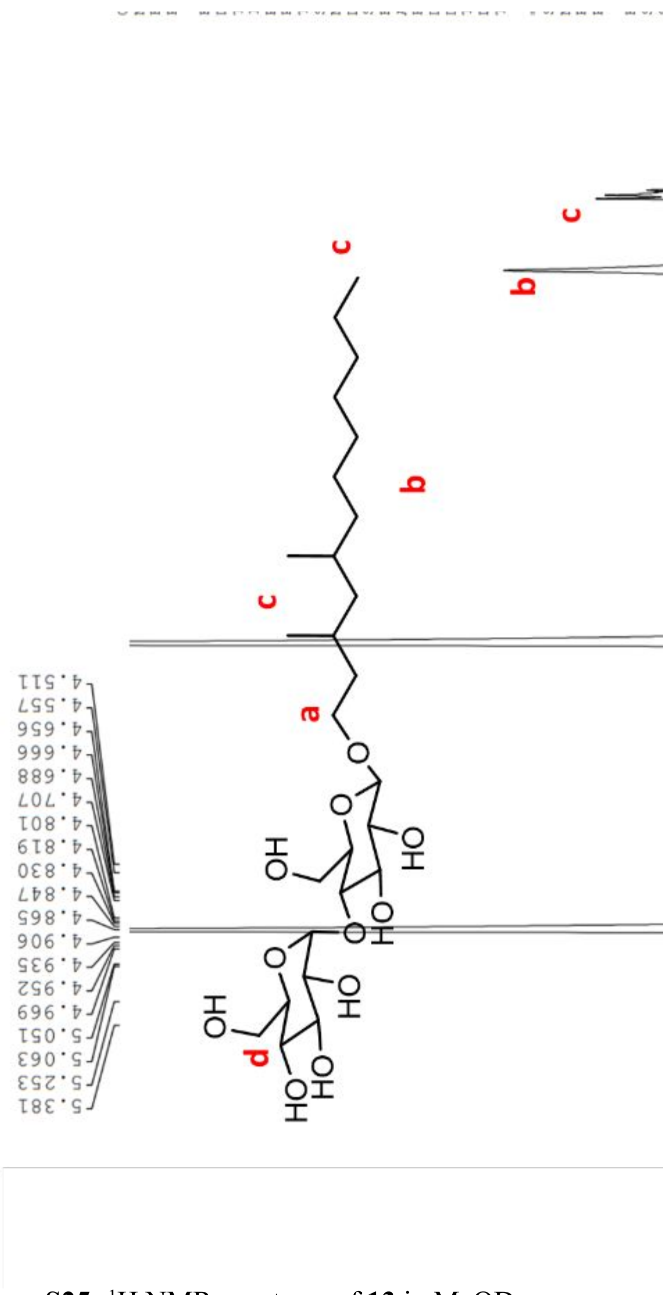

Figure S25: ^{1}H NMR spectrum of **13** in MeOD

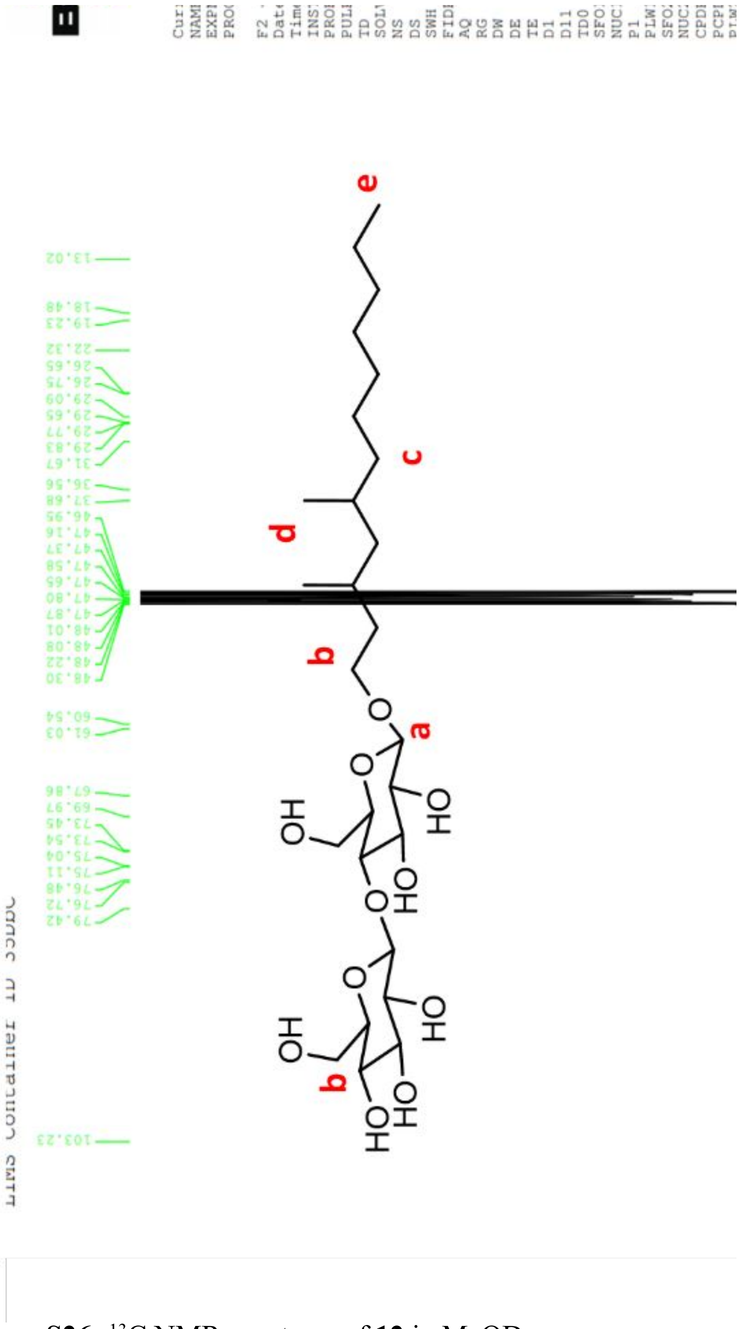

Figure S26: ^{13}C NMR spectrum of **12** in MeOD

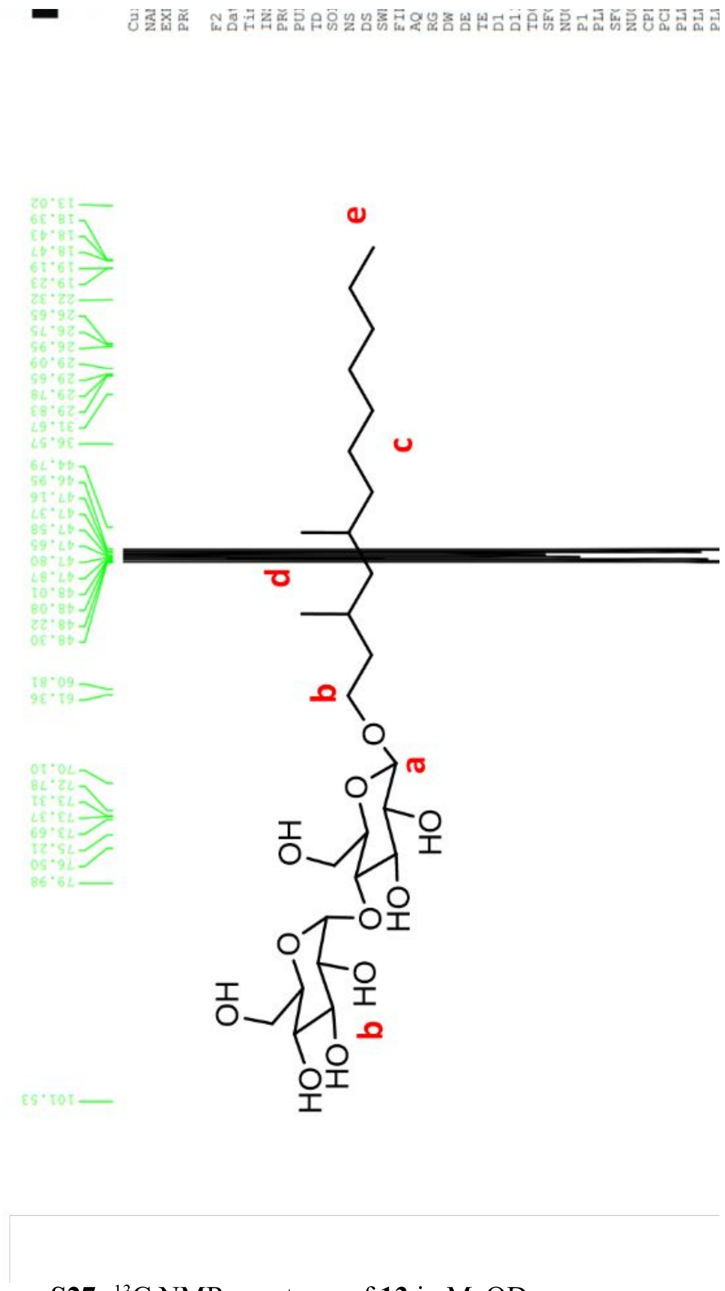

Figure S27: ^{13}C NMR spectrum of **13** in MeOD

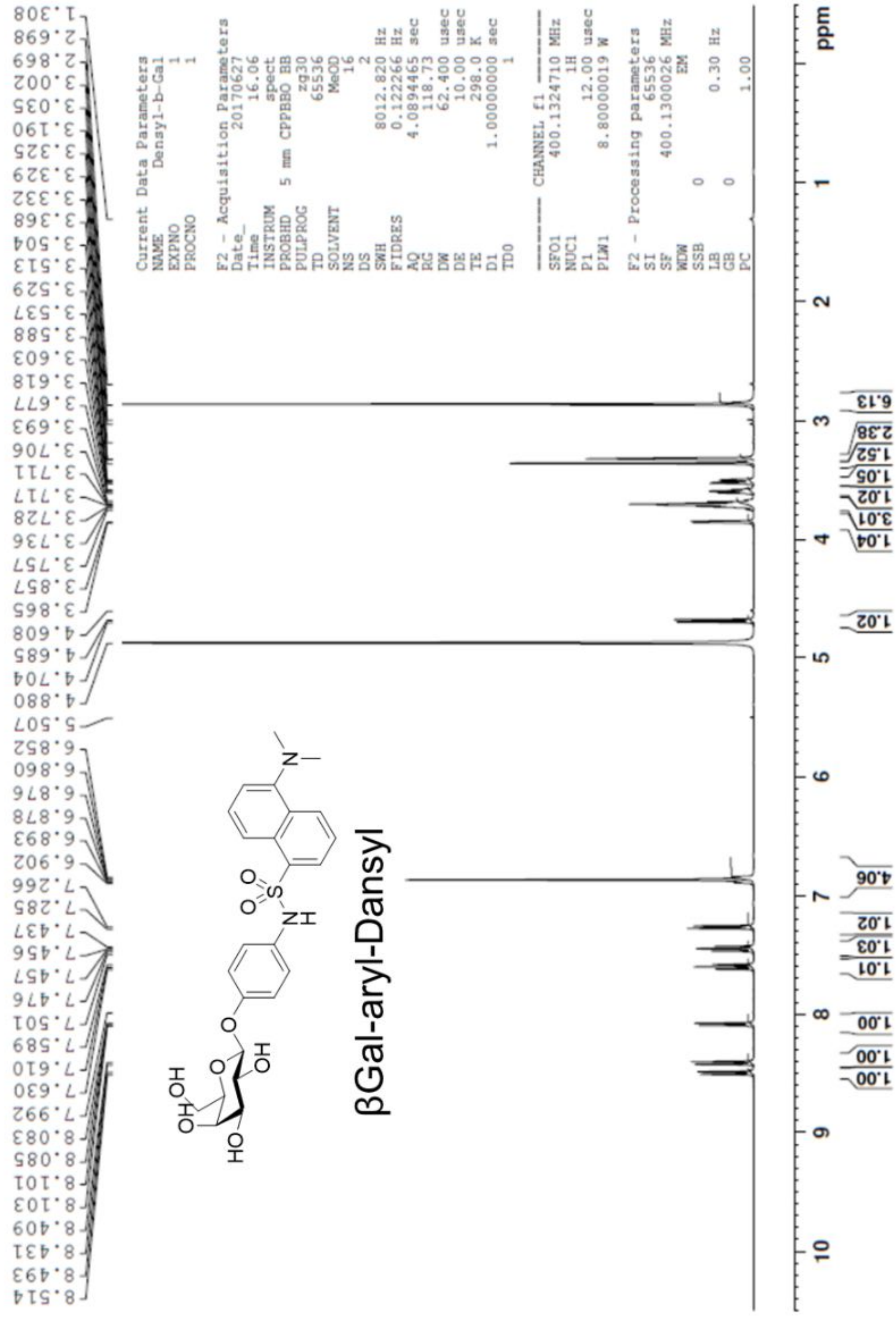

Figure S28: ^{1}H NMR spectrum of βGal-aryl-Dansyl in $CDCl_3$

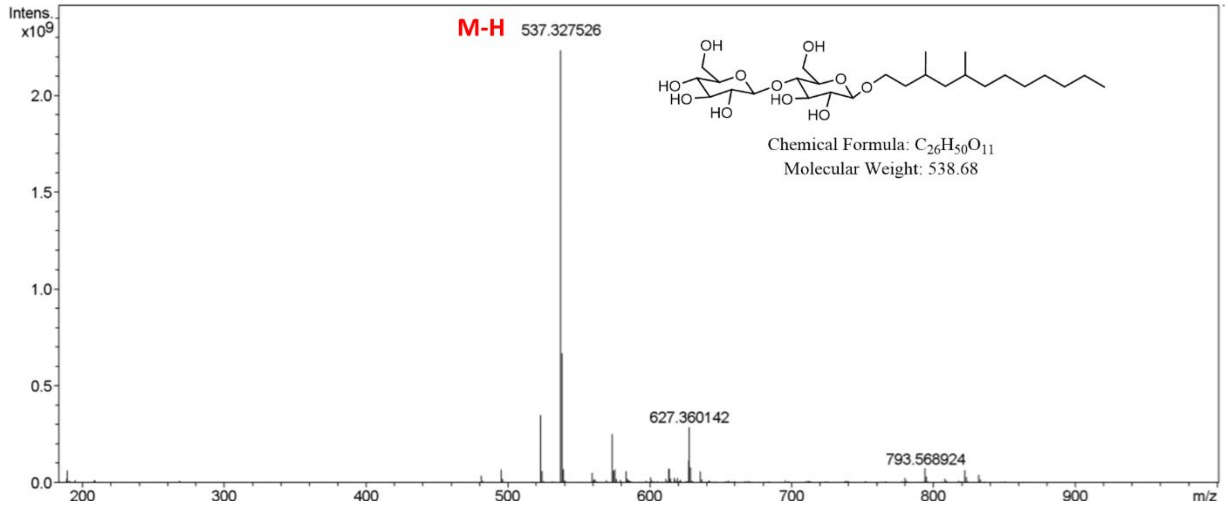

Figure S29: Mass spectroscopy by - negative-ion mode electrospray ionization of **12**

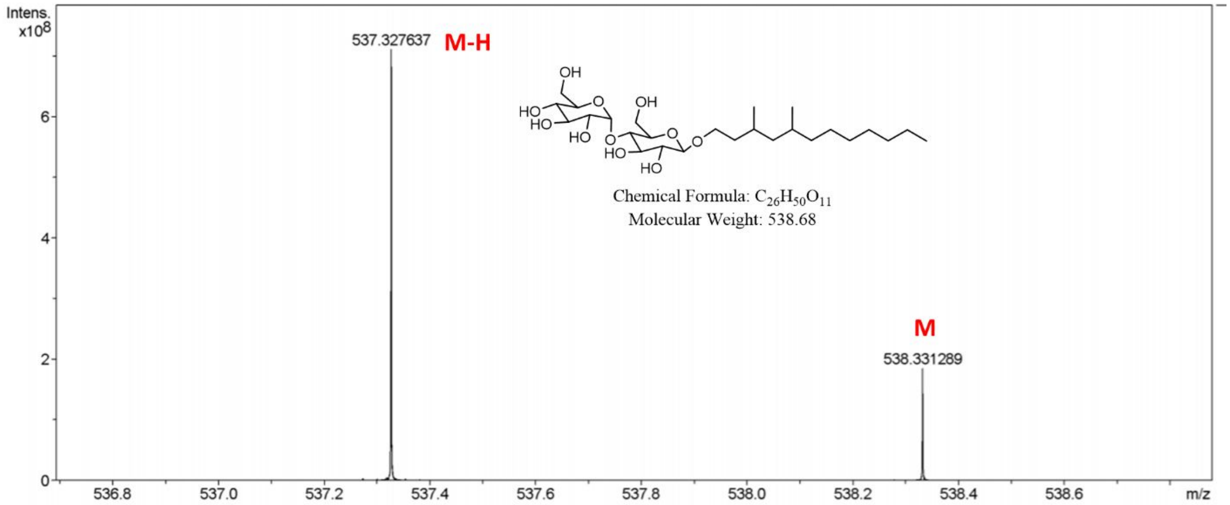

Figure S30: Mass spectroscopy by - negative-ion mode electrospray ionization of **13**

4. References

1. Singh, N.; Shetye, G. S.; Zheng, H.; Sun, J.; Luk, Y. Y., Chemical signals of synthetic disaccharide derivatives dominate rhamnolipids at controlling multiple bacterial activities. *ChemBioChem* **2016,** *17* (1), 102-111.
2. Castric, P., pilO, a gene required for glycosylation of Pseudomonas aeruginosa 1244 pilin. *Microbiology* **1995,** *141* (5), 1247-1254.
3. Horzempa, J.; Held, T. K.; Cross, A. S.; Furst, D.; Qutyan, M.; Neely, A. N.; Castric, P., Immunization with a Pseudomonas aeruginosa 1244 pilin provides O-antigen-specific protection. *Clin. Vaccine Immunol.* **2008,** *15* (4), 590-597.
4. Chibeu, A.; Ceyssens, P.-J.; Hertveldt, K.; Volckaert, G.; Cornelis, P.; Matthijs, S.; Lavigne, R., The adsorption of Pseudomonas aeruginosa bacteriophage ϕKMV is dependent on expression regulation of type IV pili genes. *FEMS Microbiol. Lett.* **2009,** *296* (2), 210-218.
5. Chua, S. L.; Liu, Y.; Yam, J. K. H.; Chen, Y.; Vejborg, R. M.; Tan, B. G. C.; Kjelleberg, S.; Tolker-Nielsen, T.; Givskov, M.; Yang, L., Dispersed cells represent a distinct stage in the transition from bacterial biofilm to planktonic lifestyles. *Nat. Commun.* **2014,** *5* (1), 1-12.
6. Rodesney, C. A.; Roman, B.; Dhamani, N.; Cooley, B. J.; Katira, P.; Touhami, A.; Gordon, V. D., Mechanosensing of shear by Pseudomonas aeruginosa leads to increased levels of the cyclic-di-GMP signal initiating biofilm development. *Proc. Natl. Acad. Sci. U.S.A.* **2017,** *114* (23), 5906-5911.
7. Persat, A.; Inclan, Y. F.; Engel, J. N.; Stone, H. A.; Gitai, Z., Type IV pili mechanochemically regulate virulence factors in Pseudomonas aeruginosa. *Proc. Natl. Acad. Sci. U.S.A.* **2015,** *112* (24), 7563-7568.
8. Siryaporn, A.; Kuchma, S. L.; O'Toole, G. A.; Gitai, Z., Surface attachment induces Pseudomonas aeruginosa virulence. *Proc. Natl. Acad. Sci. U.S.A.* **2014,** *111* (47), 16860-16865.
9. Chua, S. L.; Liu, Y.; Li, Y.; Jun Ting, H.; Kohli, G. S.; Cai, Z.; Suwanchaikasem, P.; Kau Kit Goh, K.; Pin Ng, S.; Tolker-Nielsen, T., Reduced intracellular c-di-GMP content increases expression of quorum sensing-regulated genes in Pseudomonas aeruginosa. *Front. Cell. Infect. Microbiol.* **2017,** *7*, 451.
10. O'Toole, G. A., Microtiter dish biofilm formation assay. *Journal of visualized experiments: JoVE* **2011,** (47).
11. Chua, S. L.; Yam, J. K. H.; Hao, P.; Adav, S. S.; Salido, M. M.; Liu, Y.; Givskov, M.; Sze, S. K.; Tolker-Nielsen, T.; Yang, L., Selective labelling and eradication of antibiotic-tolerant bacterial populations in Pseudomonas aeruginosa biofilms. *Nat. Commun.* **2016,** *7* (1), 1-11.
12. Kim, H.-S.; Cha, E.; Kim, Y.; Jeon, Y. H.; Olson, B. H.; Byun, Y.; Park, H.-D., Raffinose, a plant galactoside, inhibits Pseudomonas aeruginosa biofilm formation via binding to LecA and decreasing cellular cyclic diguanylate levels. *Sci. Rep.* **2016,** *6* (1), 1-10.
13. Strehmel, J.; Neidig, A.; Nusser, M.; Geffers, R.; Brenner-Weiss, G.; Overhage, J., Sensor kinase PA4398 modulates swarming motility and biofilm formation in Pseudomonas aeruginosa PA14. *Appl. Environ. Microbiol.* **2015,** *81* (4), 1274-1285.
14. Madsen, J. S.; Lin, Y.-C.; Squyres, G. R.; Price-Whelan, A.; de Santiago Torio, A.; Song, A.; Cornell, W. C.; Sørensen, S. J.; Xavier, J. B.; Dietrich, L. E., Facultative control of matrix production optimizes competitive fitness in Pseudomonas aeruginosa PA14 biofilm models. *Appl. Environ. Microbiol.* **2015,** *81* (24), 8414-8426.
15. Huang, H.; Lai, W.; Cui, M.; Liang, L.; Lin, Y.; Fang, Q.; Liu, Y.; Xie, L., An evaluation of blood compatibility of silver nanoparticles. *Sci. Rep.* **2016,** *6* (1), 1-15.
16. Stuart, M. C.; van de Pas, J. C.; Engberts, J. B., The use of Nile Red to monitor the aggregation behavior in ternary surfactant–water–organic solvent systems. **2005,** *18* (9), 929-934.

17. Hagelueken, G.; Adams, T. M.; Wiehlmann, L.; Widow, U.; Kolmar, H.; Tümmler, B.; Heinz, D. W.; Schubert, W.-D., The crystal structure of SdsA1, an alkylsulfatase from Pseudomonas aeruginosa, defines a third class of sulfatases. *Proc. Natl. Acad. Sci. U.S.A.* **2006,** *103* (20), 7631-7636.
18. Beatson, S. A.; Whitchurch, C. B.; Sargent, J. L.; Levesque, R. C.; Mattick, J. S., Differential regulation of twitching motility and elastase production by Vfr in Pseudomonas aeruginosa. *J. Bacteriol.* **2002,** *184* (13), 3605-3613.
19. Shehata, M. M.; Sayed, A. A., Genetic diversity and twitching motility of Pseudomonas aeruginosa strains isolated from different origins. *Arch. Clin. Microbiol.* **2011,** *2* (5).
20. Harvey, H.; Bondy-Denomy, J.; Marquis, H.; Sztanko, K. M.; Davidson, A. R.; Burrows, L. L., Pseudomonas aeruginosa defends against phages through type IV pilus glycosylation. *Nat. Microbiol.* **2018,** *3* (1), 47-52.
21. Kim, S.; Rahman, M.; Seol, S. Y.; Yoon, S. S.; Kim, J., Pseudomonas aeruginosa bacteriophage PA1Ø requires type IV pili for infection and shows broad bactericidal and biofilm removal activities. *Appl. Environ. Microbiol.* **2012,** *78* (17), 6380-6385.
22. Le, S.; Yao, X.; Lu, S.; Tan, Y.; Rao, X.; Li, M.; Jin, X.; Wang, J.; Zhao, Y.; Wu, N. C., Chromosomal DNA deletion confers phage resistance to Pseudomonas aeruginosa. *Sci. Rep.* **2014,** *4* (1), 1-8.
23. Joachim, I.; Rikker, S.; Hauck, D.; Ponader, D.; Boden, S.; Sommer, R.; Hartmann, L.; Titz, A., Development and optimization of a competitive binding assay for the galactophilic low affinity lectin LecA from Pseudomonas aeruginosa. *Org. Biomol. Chem.* **2016,** *14* (33), 7933-7948.
24. Caballero, A. R.; Moreau, J. M.; Engel, L. S.; Marquart, M. E.; Hill, J. M.; O'Callaghan, R. J., Pseudomonas aeruginosa protease IV enzyme assays and comparison to other Pseudomonas proteases. *Anal. Biochem* **2001,** *290* (2), 330-337.
25. Pinzon, N. M.; Ju, L.-K., Analysis of rhamnolipid biosurfactants by methylene blue complexation. *Appl. Microbiol. Biotechnol.* **2009,** *82* (5), 975-981.
26. Cao, H.; Xia, T.; Li, Y.; Xu, Z.; Bougouffa, S.; Lo, Y. K.; Bajic, V. B.; Luo, H.; Woo, P. C.; Yan, A., A multidrug resistant clinical P. aeruginosa isolate in the MLST550 clonal complex: uncoupled quorum sensing modulates the interplay of virulence and resistance. *Antimicrob. Agents Chemother.* **2019**.
